# Cardiac digital twins: a tool to investigate the function and treatment of the diabetic heart

**DOI:** 10.1186/s12933-025-02839-w

**Published:** 2025-07-18

**Authors:** Marina Strocchi, Daniel J. Hammersley, Brian P. Halliday, Sanjay K. Prasad, Steven A. Niederer

**Affiliations:** 1 National Heart and Lung Institute, Imperial College London, 72 Du Cane Road, London, W12 0NN UK; 2 King’s College Hospital NHS Foundation Trust, Denmark Hill, London, SE5 9RS UK; 3 Royal Brompton & Harefield Hospital, Guy’s & St Thomas’ NHS Foundation Trust, Sydney Street, London, SW3 6NP UK; 4 British Heart Foundation Centre of Research Excellence, School of Cardiovascular and Metabolic Medicine and Sciences, King’s College London, London, UK; 5 The Alan Turing Institute, The British Library, 96 Euston Road, London, NW1 2DB UK

**Keywords:** Diabetes, Heart, Model, Cardiac computational model, Digital twin, Anti-diabetic treatment, Cardiovascular outcome trials, In-silico trials

## Abstract

Diabetes increases the risk of cardiovascular disease (CVD) due to its multi-scale and diverse effects on cardiomyocyte metabolism and function, the circulation, and the kidneys. The complex relationship between organ systems affected by diabetes and associated comorbidities leads to challenges in estimating cardiovascular risk and stratifying optimal treatment strategies at the individual patient level. Most recently, sodium-glucose transport protein 2 (SGLT2) inhibitors and glucagon-like peptide-1 (GLP1) receptor agonists have been shown to offer substantial cardiac benefits. However, the direct or indirect mechanisms through which these agents protect the heart remain unclear, posing a challenge to patient selection. Amidst a growing burden of diabetes and increased therapeutic armamentarium, there is an important unmet need to develop more precise methods and technologies to understand the effects of diabetes and anti-diabetic treatment on the heart with faster timelines than conventional randomised controlled trials. Cardiac computational models could be used to improve our understanding of the cardiac changes in diabetes and to predict how a patient’s heart will respond to anti-diabetic treatment. In this review, we provide an overview of current cardiac computational models to investigate the diabetic heart and the cardiac effects of anti-diabetic treatment. We discuss how multi-scale and multi-physics models could be applied in future to support the development of novel therapeutic approaches and further improve the treatment of diabetic patients with different CVD risk.

**Graphical abstract:**

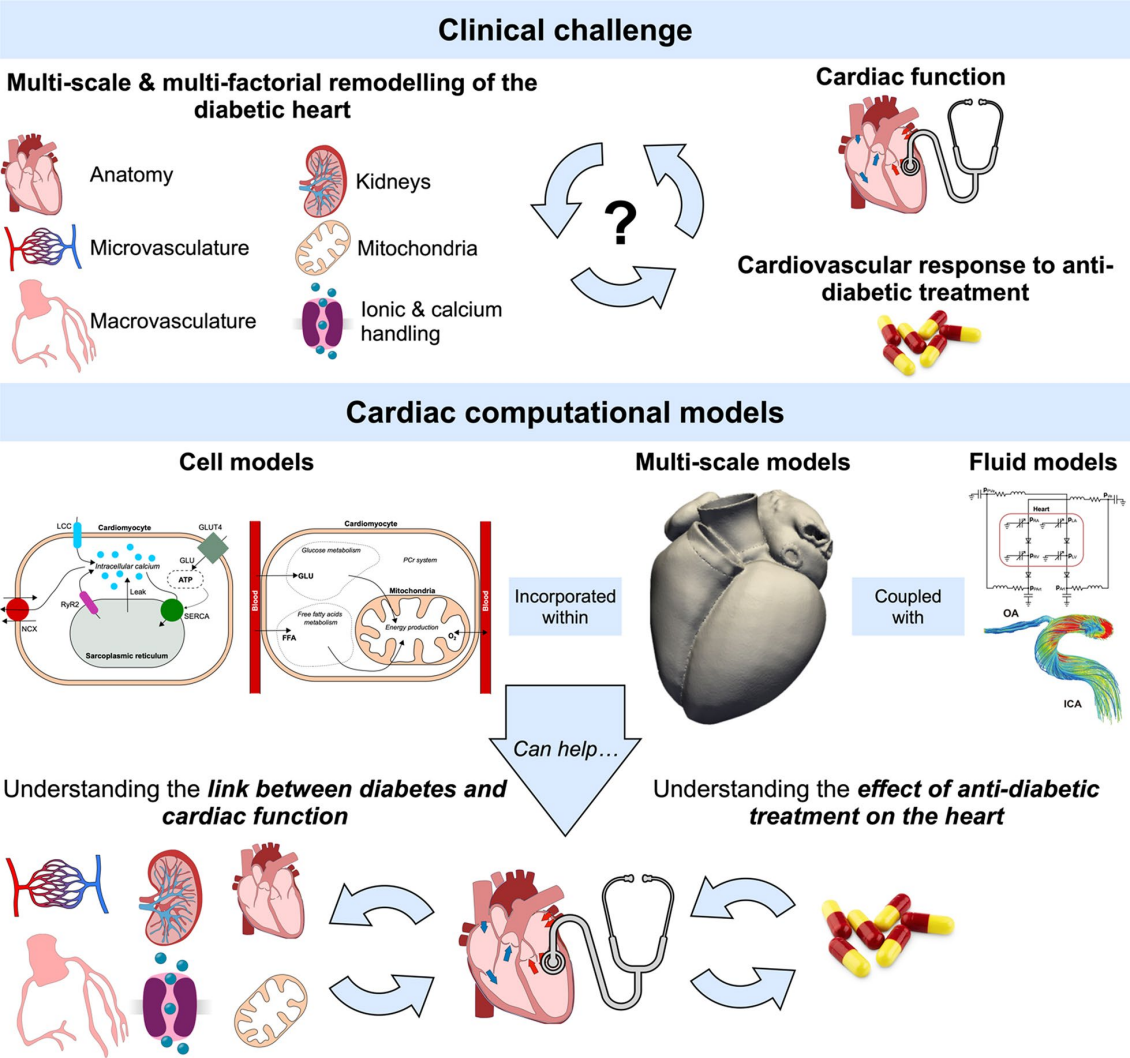

**Supplementary Information:**

The online version contains supplementary material available at 10.1186/s12933-025-02839-w.

## Research insights

**What is currently known about this topic?**

- Diabetes increases the risk of cardiovascular disease through a plethora of multi-scale (from the single protein to the whole heart) and multi-organ (heart, pancreas, and kidneys) remodelling mechanisms.
- Anti-diabetic drugs have been shown to provide cardiac benefits, but the underlying mechanisms remain unclear, requiring the development of new methodologies to understand their effects on the heart. Cardiac computational models are advanced digital representations of the heart that simulate its metabolism, electrophysiology, mechanics, and interaction with the circulatory system.

**What is the key research question?**

- How can cardiac computational models improve the cardiovascular outcome of diabetic patients?

**What is new?**

- This is the first review describing the cardiac computational models applied to diabetes and anti-diabetic treatment, from myocyte metabolism, electrophysiology, and calcium handling to blood flow and the whole cardiovascular system. We discuss the potential of more complex multi-scale electromechanics and perfusion models to incorporate multiple mechanisms of interaction between diabetes and the heart, and how they could be used to understand how sex and comorbidities such as obesity and heart failure affect cardiovascular outcomes in diabetes.

**How might this study influence clinical practice?**

- This review outlines how cardiac computational models could be applied in future to support more efficient development of novel therapeutic approaches and further improve the treatment of diabetic patients with different cardiovascular risk.

## Introduction

The number of people with diabetes has increased from 200 to 830 million between 1990 and 2022, indicating an alarming rise in prevalence accompanied by increased diagnosis and awareness [1]. Both type 1 and type 2 diabetes increase the risk of cardiovascular disease (CVD), including hypertension [2], coronary artery disease [3], heart failure (HF) [4], and arrhythmias [5]. Patients with type 1 diabetes are typically diagnosed at a younger age, resulting in longer disease duration, while type 2 diabetes often presents later in life. Early-onset type 2 diabetes, however, is associated with a higher CVD burden compared to both type 1 diabetes and late-onset type 2 diabetes [6]. This disparity in disease onset and duration complicates direct comparisons of CVD incidence between diabetes types. Early-onset type 2 diabetes demonstrates more aggressive progression and earlier development of both microvascular and macrovascular complications, with a significantly increased risk of myocardial infarction and stroke compared to later-onset cases [6]. A sub-group of patients with diabetes exhibit abnormal myocardial structure and performance in the absence of other cardiac risk factors, such as coronary artery disease, hypertension or significant valvular disease, a pathology referred to as *diabetic cardiomyopathy* [7–9]. In 1972, Rubler et al. [10] provided the first clinical characterisation of diabetic cardiomyopathy to report post-mortem data from four diabetic patients who died of HF without evidence of cardiovascular risk factors other than diabetes. This terminology was confirmed by the Heart Failure Association in 2018 [11], which stated the most commonly accepted definition of diabetic cardiomyopathy as a myocardial dysfunction which occurs in the absence of all other CVDs. However, consensus about its definition and existence is still lacking [12]. In addition, there are important sex differences to consider. Diabetic women have a higher risk of developing CVD compared to diabetic men, although the reasons behind this difference are poorly understood [13]. CVD is the leading cause of death among diabetic patients [14]. Understanding when and how diabetes accelerates cardiac dysfunction and developing a more personalised approach to stratifying cardiovascular risk among patients with diabetes has the potential to improve the treatment and management of diabetic patients.

**Fig. 1:**
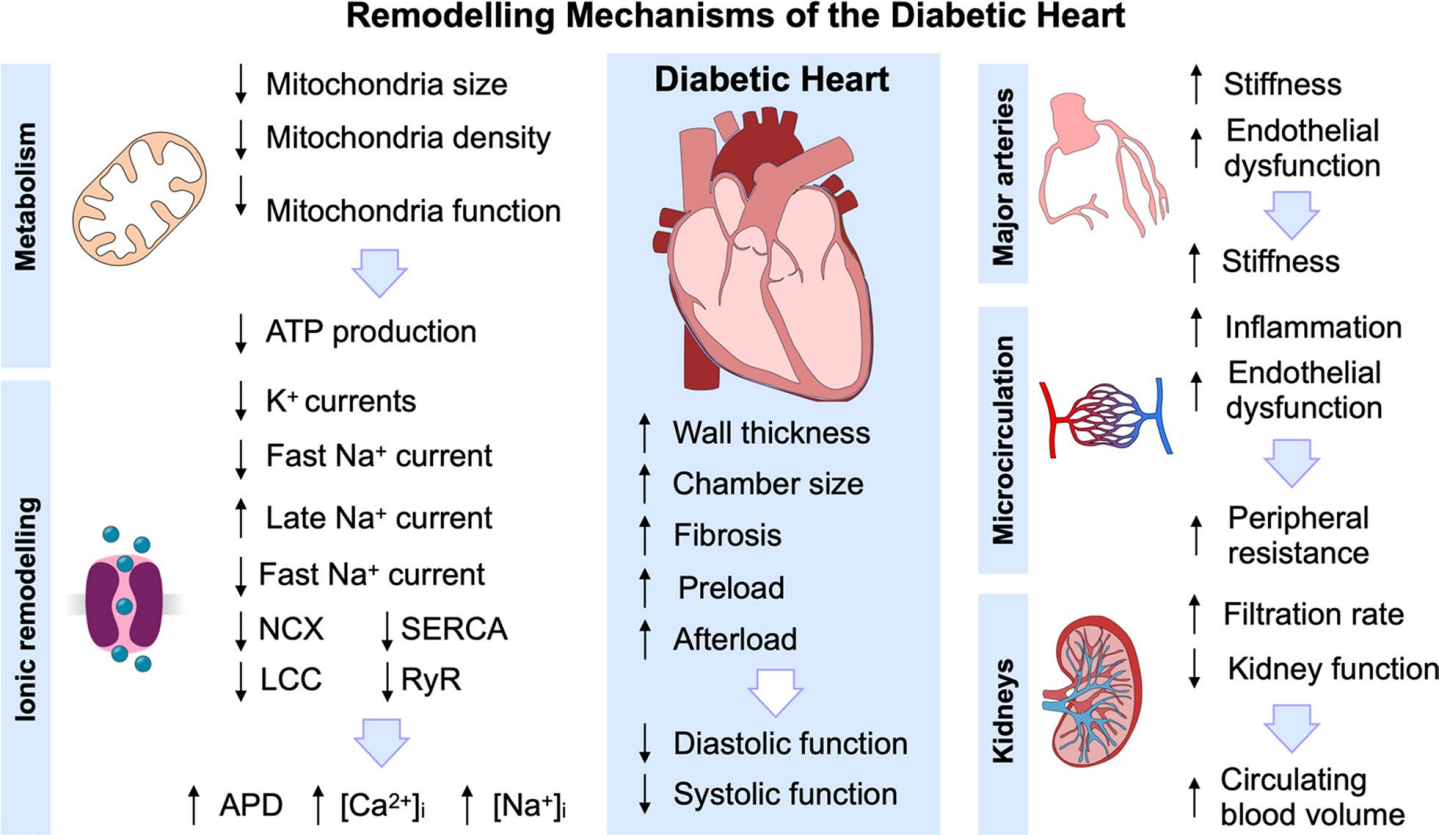
Remodelling mechanisms of the diabetic heart. Diabetes affects cardiac function through a plethora of multi-scale remodelling mechanisms, from the single protein and cell up to the whole circulatory system level. In cardiomyocytes, high glucose causes a metabolic shift leading to mitochondrial remodelling and therefore reduced ATP production. The diabetic cell also undergoes ionic channel remodelling, which causes increased APD, and intracellular calcium and sodium overload, contributing to diastolic dysfunction. Diabetes also causes endothelial dysfunction and inflammation, which increases the stiffness in the major arteries and in the microcirculation, leading to increased peripheral resistance. Finally, hyperglycaemia causes increased kidney filtration rate, decreased kidney function, and therefore increased circulating blood volume. All these remodelling mechanisms contribute towards anatomical changes in the heart, diastolic dysfunction, systolic dysfunction and, ultimately HF. *ATP* adenosine triphosphate; *K*^+^ potassium; *Na*^+^ sodium; *NCX* sodium-calcium exchanger; *SERCA* sarcoplasmic/endoplasmic reticulum calcium-ATPase; *LCC* L-type calcium channels; *RyR* ryanodine receptors; *Ca*^*2*+^ calcium

Prolonged hyperglycaemia leads to a wide range of changes in the diabetic heart and circulation, from the single protein [15–29] to the whole circulatory system scale and other organs (Fig. 1) [30–32]. The myocytes of patients with chronic high blood glucose are characterised by impaired glucose uptake inside the cell due to malfunction and under-expression of the glucose transport proteins [8]. This causes a decreased glucose utilisation, upregulated free fatty acids (FFAs) metabolism and lipotoxicity, that is the accumulation of lipids and lipid intermediate metabolites (molecules produced by FFA metabolism) [7]. These substances together with the increased production of advanced glycation end-products (AGEs) also triggered by hyperglycaemia contribute towards inflammation [8, 33]. Hyperglycaemia also leads to mitochondrial dysfunction and consequent increased production of reactive oxygen species (ROS) and oxidative stress, which further promotes the production of AGEs and therefore inflammation [33, 34]. Inflammation, oxidative stress and altered metabolism characteristic of diabetes lead to collagen formation, myocyte apoptosis, hypertrophy and necrosis and therefore have severe consequences on the heart and the circulatory system [8, 33, 34].

At the single cardiomyocyte level, because FFAs metabolism is less efficient than glucose metabolism and triggers an inflammatory response, high glucose levels cause reduced energy production, decreased adenosine triphosphate (ATP) availability, mitochondrial remodelling and dysfunction, and inflammation [15]. Because calcium handling and cardiac contractility heavily rely on energy consumption, low ATP availability within the cell causes a cascade of remodelling mechanisms that can result in diastolic and systolic dysfunction and ultimately HF. In addition, diabetic cardiac myocytes have been reported to undergo ionic remodelling mechanisms. Animal studies on isolated ventricular myocytes have shown that the transient outward potassium current is decreased in diabetic rats [18, 26], and that the fast and slow and rapid rectifier potassium currents are reduced in diabetic rabbits [16, 17]. Similarly, the ultra-rapid outward potassium current is reduced in isolated atrial myocytes from diabetic mice [20]. Measurements performed on diabetic rodents have demonstrated that the fast [20] and late sodium [28, 29] currents are also altered in atrial and ventricular diabetic myocytes. Finally, the expression and/or function of several proteins involved in calcium handling are reduced in diabetes, including sodium-calcium exchanger (NCX) expression [21], L-type calcium current [17, 26, 27], and ryanodine receptor (RyR) [21, 27] and sarcoplasmic/endoplasmic reticulum calcium ATPase (SERCA) [21, 23] activity. These mechanisms all potentially contribute towards prolonged action potential duration (APD), intracellular sodium and calcium overload and loss of cell contractility, which can in turn result in increased arrhythmia risk and depressed cardiac function. At the whole-heart level, this in combination with collagen accumulation triggered by the inflammatory response [33] translates into concentric remodelling [35, 36], fibrosis [37–39], atrial dilation [40], diastolic dysfunction [40], decreased systolic shortening and contractile dysfunction [37], and hypertrophy [39]. Diabetes causes micro- and macro-vascular disease, initiated by hyperglycaemia-induced endothelial dysfunction, increased ROS production and inflammation [41, 42]. High glucose initiates a cascade of remodelling mechanisms that result in decreased vasodilatory capacity of the microvascular bed [42], increased risk of plaque formation in the major arteries (e.g., carotid and coronaries) [43], and damaged arterial baroreflectors (mainly located in the aortic arch and the carotid sinuses), contributing towards impaired vasodilation in response to changes in blood pressure [44]. These remodelling mechanisms affect the circulatory system, manifested as poor systemic perfusion of peripheral organs, for example in the eyes therefore increasing the risk of vision loss [45], and impaired kidney function [31]. Poor kidney function is a common comorbidity of diabetes [31], which further contributes towards cardiac deterioration due to increased blood volume and preload. The multi-scale and multi-factorial nature of all the remodelling mechanisms of the diabetic heart, combined with the increased incidence of traditional CVD risk factors in the diabetic population (for example, obesity, low physical exercise, hypertension, and dyslipidaemia)[46], make it challenging to distinguish the relative contribution of each towards cardiac dysfunction.

The complexity of diabetes and its interaction with multiple comorbidities make it difficult to select the best treatment strategy for each patient, constituting an important unmet need. Furthermore, because some drug classes have been shown to increase the risk of HF hospitalisation, new anti-diabetic drugs must undergo cardiovascular outcome trials (CVOT) to test cardiac safety [47]. Whilst CVOTs are regarded as the gold standard, they are expensive, time-consuming and with a long time lag and attendant delay in clinical deployment of new anti-diabetic drugs [47]. Novel technologies such as cardiac computational modelling could help streamline this process and provide mechanistic insight into the molecular basis of the apparent cardioprotective effects demonstrated by newer agents such as sodium-glucose transport protein 2 inhibitors (SGLT2i) and glucagon-like peptide-1 receptor agonists (GLP1-RAs), which to date remain incompletely explained.

## Overview of computational models of the heart and cardiovascular system

**Fig. 2:**
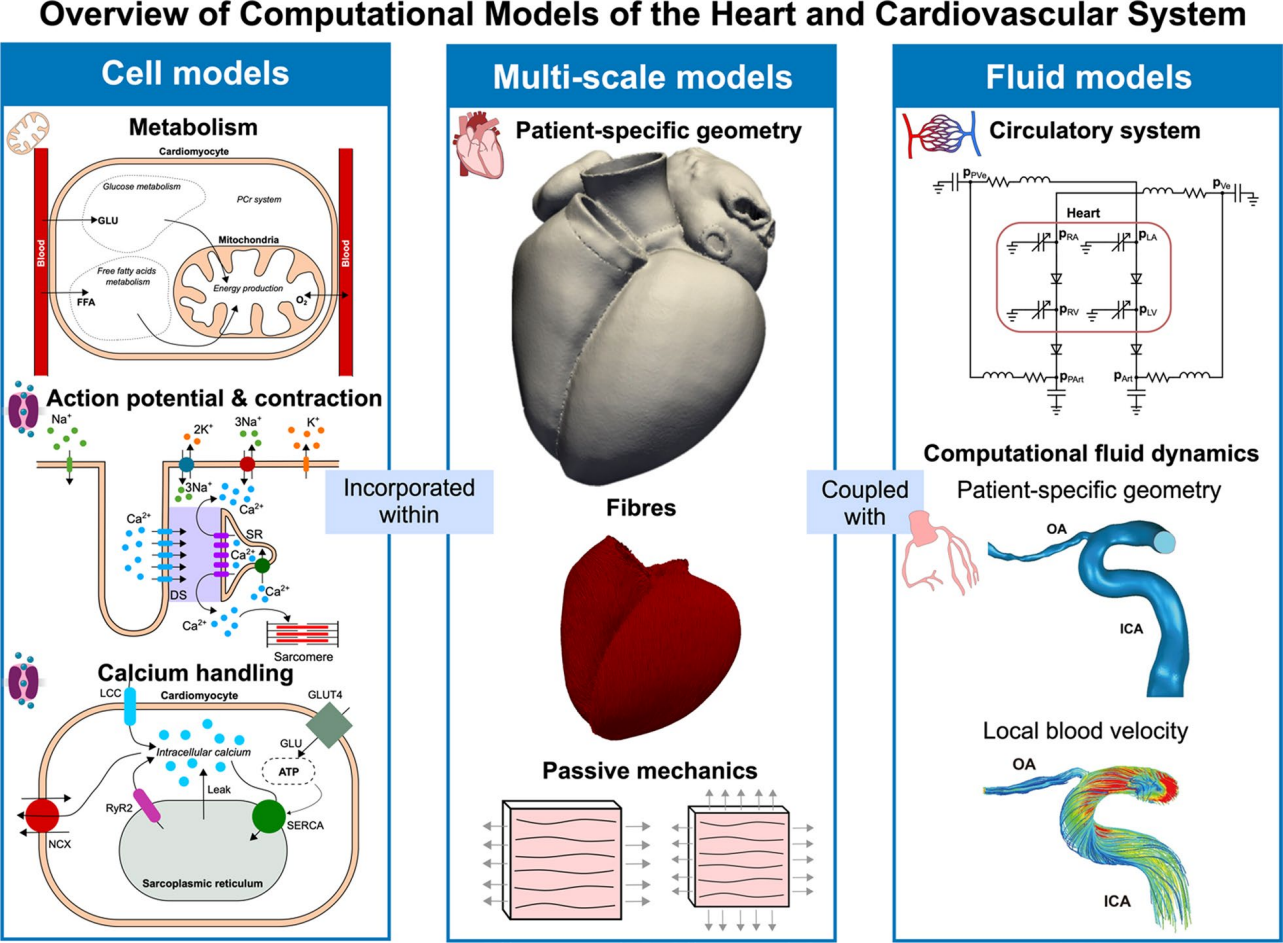
Overview of computational models of the heart and cardiovascular system. Models for cardiomyocyte metabolism simulate energy production from FFAs and glucose within the cytosol and the mitochondria. Action potential and myocyte contraction models simulate different ion species moving in and out of the cell generating the action potential, the intracellular calcium transient and contraction. Models for calcium handling can be used to focus on cellular processes responsible for calcium absorption and excretion, and the effect of ATP production from glucose entering the cell. These different types of cell models can be incorporated in multi-scale frameworks where the patient-specific geometry of the heart is generated from imaging data, and the fibre arrangement is included with rule-based methods. Finally, a multi-scale electromechanics model of the heart can be coupled with a model for the circulatory system to simulate the preload and the afterload of the heart. More complex computational fluid dynamics (CFD) simulations use a patient-specific geometry of the patient’s vessels to compute local blood velocity. The CFD figures of the ophthalmic and internal carotid artery (OA and ICA) were adapted from “Ophthalmic artery changes in type 2 diabetes with and without acute coronary syndrome” by Wu et al.[48], published under CC BY 4.0 https://creativecommons.org/licenses/by/4.0/. *GLU* glucose, *FFA* free fatty acids, *O*_*2*_ oxygen, *PCr* Phosphocreatine, *Na*^+^ sodium, *K*^+^ potassium, *Ca*^*2*+^ calcium, *SR* sarcoplasmic reticulum, *DS* dyadic space, *LCC* L-type calcium channels, *NCX* sodium-calcium exchanger, *RyR2* ryanodine receptor, *SERCA* sarcoplasmic/endoplasmic reticulum calcium ATPase, *OA* ophthalmic artery, *ICA* internal carotid artery

There are different types of computational models of the heart and cardiovascular system, simulating different scales (protein, cell or whole organ) and physics (electrophysiology, mechanics or fluid dynamics). Below, we provide a brief overview of these models (Fig. 2). In the Supplement, we provide a more detailed overview of computational models of the heart and cardiovascular system that contextualises the computational frameworks described below.

Models for cardiomyocyte metabolism represent the biochemical and biophysical processes leading to energy production and consumption [49–51]. They combine metabolic pathways and cellular energetics to simulate aerobic respiration and mitochondrial function through glucose and FFAs metabolism. These models can also include ion transport across the cell membrane and within the cell (from the cytoplasm to the sarcoplasmic reticulum) to account for energy-demanding processes, such as cell contraction or active ionic transport, directly affecting ATP consumption [49, 50]. This type of framework can be used to quantify how cardiac cells adapt to energy consumption and demand in normal and diseased conditions where cellular metabolism is shifted (e.g., diabetes) by altering, for example, model parameters to mimic increased or decreased substrate availability or usage.

Action potential models represent the electrical activity of a cardiomyocyte by simulating the movement of different ion species (mainly sodium, potassium, and calcium) across the cell membrane. These models are typically calibrated using experimental measurements for the action potential, calcium transient as well as voltage-clamp data, and have been developed for different species [52–56] (rat, rabbit, human) and cell types [52, 53, 57]. They can be used to quantify the arrhythmogenic properties of different drugs and to link genetic mutations or alterations in protein function to action potential abnormalities (e.g., APD prolongation). Action potential models can also be coupled with models for cardiomyocyte contraction [56, 58, 59], which represent the kinetic processes leading to active tension development, and sarcomere and cellular contraction. In these coupled models, changing parameters representing protein density and/or function to represent a specific disease phenotype allows linking protein function to the contractile properties of the cell.

Action potential models can be incorporated into tissue and whole-organ models to investigate how protein and cellular properties affect heart electrophysiology and mechanics [60]. Patient-specific anatomical models can be generated from magnetic resonance imaging (MRI) or computed tomography (CT) datasets [61]. Myofiber orientation is incorporated in these models to represent the cardiac tissue microstructure using rule-based methods or diffusion tensor MRI data [62–66]. The monodomain or the bidomain model [67], based on the reaction–diffusion equation, can then be used to represent tissue voltage and extracellular potential changes over time. The heart model can also be embedded in the torso to simulate 12-lead electrocardiograms (ECGs), to quantify the effect of cellular properties on clinically relevant quantities, such as the QRS duration and the QT interval. Models for cell contraction can also be included in whole-heart and tissue mechanics frameworks to simulate the patient’s heart mechanical contraction. In these models, the anisotropic, passive mechanical properties of the tissue are represented with passive material laws that mimic the stresses developed in the myocardium as it deforms [68–70]. The cellular active tension generation then drives whole-heart contraction to simulate the patient’s heart deforming as it beats. Whole-heart electromechanics models can represent multi-scale cardiac function, from the single protein up to the cell and the organ, and therefore have the potential to link them to emergent cardiac function in normal and diseased patients.

Similar to whole-heart electromechanics models, computational fluid dynamics (CFD) rely on patient-specific geometries extracted from medical images [71, 72]. These types of models however make use of the geometry of the blood pool of the cardiac chambers or large vessels rather than the myocardium. These anatomical models are then used to solve the fluid dynamics (Navier–Stokes) equations, describing the motion of blood inside static or dynamically deforming vessels. CFD models can be used to investigate blood flow in normal or diseased vessels (e.g., in the presence of stenosis or aneurysms). CFD frameworks can also be coupled with perfusion models, which typically employ an idealised model of fluid flow through a porous medium (described by Darcy’s Law) [73–76]. This type of framework allows quantifying blood flow distribution and oxygen delivery to different tissues in the body (e.g., the myocardium) through the microcirculation while accounting for the effects of both macro and microvasculature properties.

Electromechanics and CFD models are highly detailed, but they require high computational costs (in the order of hours on a supercomputer), hindering their clinical applicability. Circulatory system models, also called lumped parameter models, constitute a fast and more efficient alternative, offering a lumped representation of the heart and cardiovascular system [77–79]. This type of framework assumes that each component behaves as a simplified circuit with specific properties (resistance, compliance, and inertia), and pressure and flow are simulated as voltage and electrical current. The heart is typically represented by a time-varying elastance model to simulate the periodic contractile function of the heart. This more efficient approach allows simulating the heart and cardiovascular system over days or weeks [80, 81], and can therefore be used to predict long-term rather than only acute response to treatment. These models are also very flexible, as they can be adapted to add a more detailed representation of the compartment or compartments of interest. For instance, the simplified heart model could be replaced with a whole heart electromechanics framework to provide a detailed simulation of the electrical activity and of the contraction of the heart, while the lumped parameter model provides a physiological representation of the preload and afterload of the cardiac chambers [60]. These models can link emergent cardiac function (e.g., ejection fraction, systolic and diastolic blood pressure) to cellular, tissue and circulatory system properties, making them suitable for studying complex multi-scale and multi-organ pathologies like diabetes. However, they are complex and computationally expensive to create, run and analyse.

Recently, computational heart and cardiovascular models have been used in *in-silico trials*, where computer simulations predict clinical trial outcomes by testing drugs, devices, and treatments on virtual patients that replicate real physiology [82]. To generate virtual patients, in-silico trials can use different techniques, including digital twinning, where the model parameters are calibrated to represent a specific, real patient in the cohort of interest, or statistical populations, where the model parameters are varied so that the statistical distribution simulated biomarkers (e.g., systolic blood pressure, diastolic blood pressure, ejection fraction) match the statistical distribution of the biomarkers from the real patient population of interest. Once the virtual patients are generated, the effect of the medical therapy is simulated on each, and the virtual patients’ response is predicted with the model. In-silico trials are powerful tools that have the potential to predict the outcome of a clinical trial; provide in-silico data to assist power calculations and optimise patient recruitment; compare the effect of different treatments on the same virtual patients; and investigate the effects of medical therapies on different patient populations.

Cardiac computational modelling and in-silico trials are a fast-growing technology, increasingly used in cardiology applications to better stratify patients and improve response to therapy [83], and they have the potential to become a new quantitative method for cardiac disease diagnosis and management [84].

In this review, we will describe the cardiac computational models that have been used for diabetes applications. The models have been divided into four categories: (1) *metabolism*, describing energy production and consumption within a cardiomyocyte; (2) *electrophysiology and calcium handling*, simulating the biochemical reactions leading to the cardiac action potential and calcium flux in and out of the cell; (3) *blood flow*, simulating the circulatory system and/or blood flow through the systemic and the coronary arteries; (4) *in-silico trials*, where models are used to investigate the effect of anti-diabetic drugs on virtual patient populations. In the discussion, we highlight the potential of more advanced multi-scale, whole-heart computational models and how these can contribute towards understanding the link between diabetes and the heart and improving the treatment and management of diabetic patients with different CVD risk. In the Supplement, we provide a table with all the computational studies that have been used to investigate the effects of diabetes and anti-diabetic treatment on the heart.

## Modelling the metabolism of diabetic cardiomyocytes

Cardiac cells are very energy-demanding as their function relies on the use and production of energy in the form of ATP. ATP is used for a multitude of processes within the heart, from transporting ions across the cell membrane or from the cytosol into the sarcoplasmic reticulum (calcium re-uptake by SERCA) to contractile force generation in the sarcomere. This high energy demand means that the heart is also very flexible in its energy production, to be able to adapt to different conditions where energy production needs to be increased (e.g., exercise) or decreased (e.g., sleep). Normal myocytes primarily produce energy from FFA (40–60%) and glucose (20–40%) oxidation [85]. In the diabetic heart, insulin resistance downregulates the transport proteins responsible for glucose uptake in the cell. This causes a decreased availability of glucose and a consequent increase in FFA oxidation [86, 87]. Although this is necessary to maintain energy levels and cardiac contractility, it also reduces the metabolic flexibility of the heart. FFA oxidation consumes more oxygen, it can lead to mitochondrial uncoupling, where energy is dissipated rather than made available to the cell, and it creates more reactive oxygen species, therefore leading to inflammation [15, 85]. Furthermore, increased lipid uptake within the cell leads to lipotoxicity (the accumulation of unmetabolised lipids inside the cell), which promotes inflammation and apoptosis [85]. All these mechanisms promote fibrosis deposition, myocyte hypertrophy and, due to the lack of metabolic flexibility, increased risk of myocyte injury and inability to increase energy production on demand [15]. This drop in cardiac efficiency in the diabetic heart has been measured through reduced phosphocreatine and ATP ratio (PCr/ATP) in diabetic hearts compared to controls [86, 88] and has been linked to impaired relaxation (diastolic dysfunction), hypertrophy and ultimately left ventricle (LV) systolic dysfunction and HF [15]. However, how diabetes-induced metabolic dysfunction leads to cardiac function deterioration remains unclear. Understanding how metabolic and mitochondrial dysfunction relate to reduced cardiac efficiency and contractility is vital to improve the treatment and management of diabetic patients, and to prevent the progression to HF. Below, we describe the models published to date that have been used to investigate the metabolism of diabetic cells and to understand how alterations induced by diabetes on metabolism, mitochondrial structure and function affect cellular energy production and availability. Although these models account for only a few of the complex and multi-factorial metabolic remodelling mechanisms induced by diabetes, they still provide mechanistic insight and quantification of the consequences of diabetes on myocyte function.

Models for cardiac metabolism have been developed and applied to study the consequences of metabolic and mitochondrial alterations caused by diabetes [51, 89–92]. Zhou et al. [93] used a model for cardiac energy metabolism including the mitochondria and the cytosol to investigate the effect of increased energy expenditure in diabetic conditions under different levels of substrate availability in the blood. A model for oxidative phosphorylation was used to quantify the contribution of mitochondrial dysfunction and/or hypoxia towards a decrease in PCr/ATP, indicating a mismatch between energy supply and demand [89]. Cortassa et al. [51] combined a model for mitochondrial respiration with data collected from mitochondria isolated from healthy and type 1 diabetes guinea pigs to relate lipid concentration to oxygen consumption and were able to demonstrate that high lipid concentrations impair mitochondrial function and lead to inefficient energy production. Cortassa et al. [92, 94] also combined metabolomics data collected from healthy and diabetic mice in combination with a model for fluxes through the metabolic network (fluxome) to quantify differences in metabolism between the diabetic and the healthy heart, showing that glucose degradation pathways are reduced in diabetes [94].

Models for metabolism have also been coupled with electron microscopy data to quantify the effects of mitochondrial morphology changes on energy production and distribution [90, 91]. Jarosz et al. [91] measured mitochondrial size and density in healthy and diabetic rats, demonstrating that mitochondria in diabetic cardiomyocytes are smaller and less dense, and they tend to form bigger clusters [91]. The authors then performed simulations with a mixed-compartment model of energy transfer between mitochondria and myofibrils in myocytes to quantify the effects of altered mitochondrial morphology on energy production. The model showed that mitochondrial clustering can partly compensate for impaired energy production typical of the diabetic heart by increasing ATP availability in the cytosol. Ghosh et al. [90] extended this model. Using two-dimensional (2D) electron microscopy image slices from healthy and diabetic rat myocytes, they were able to segment out the mitochondria and myofibrils. These structures were then used to generate 2D finite element models that simulated local metabolite transport, production and consumption within the cell. The model showed that the irregular arrangement of myofibril and mitochondria of diabetic cells leads to impaired metabolite transport, leading to low energy availability in the myofibrils. These results have the potential to link mitochondrial remodelling in the diabetic cell to reduced myocardial contractility.

## Modelling electrophysiology and calcium handling in diabetic cardiomyocytes

Diabetic patients are at increased risk of developing arrhythmias [5, 36]. However, the mechanisms through which this happens remain debated. Diabetes has been shown to alter the function of a wide range of ion channels and pumps, from reduced potassium [16–19, 23–25] and fast sodium [19, 20] currents, to increased late sodium currents [28, 29], all contributing towards increased intracellular sodium concentration and APD prolongation. Altered calcium handling has also been reported as a hallmark of diabetic cardiomyocytes and has been attributed to altered NCX expression and function [21, 22], decreased L-type calcium current [17, 26, 27], decreased RyR expression [21, 27], and decreased SERCA expression [21, 23]. These mechanisms lead to raised diastolic calcium levels that are a potential contributor to diastolic dysfunction and HF and can decrease calcium transients [95] and therefore reduce myocardial contractility. Alterations in ionic currents and calcium handling in diabetes are reported by several studies, sometimes with conflicting results depending on the species or the diabetes animal model used in the experiments, making it difficult to translate these findings to patients and into clinical practice. Although cardiac computational electrophysiology and calcium handling models constitute a simplification of the intricate mechanisms linking diabetes and cellular function, they can be used to quantify the effect of the diabetes-induced remodelling mechanisms on cellular function, by altering diabetes-induced ion channel remodelling derived from experimental measurements and quantifying the consequent changes in the action potential. This can serve as a tool to bridge the gap between animal studies and arrhythmia risk quantification in diabetic patients.

### Action potential models for diabetic cells

The sinoatrial node (SAN) is the primary cardiac pacemaker, and the autonomic nervous system modulates its function. Bassil et al. [96] used a network model to link sympathetic and parasympathetic activity to the function of the SAN in mice myocytes, showing that pulmonary vein ganglia function, known to play a role in arrhythmia initiation and maintenance, was altered in diabetes and could contribute to heart rhythm irregularities. Action potential models for mouse and human SAN cells have also been used to investigate whether intracellular sodium modulates the SAN pacemaker activity [97]. By altering the NCX and the sodium–potassium pump in the model, Morotti et al. [97] were able to replicate increased intracellular sodium concentration observed in diabetic cardiomyocytes and to show that it can lead to altered automaticity and firing rate. Finally, a model for the human SAN action potential inclusive of sympathetic and parasympathetic stimulation has been used to show that hypoglycaemia and hypokalaemia can significantly decrease the heart rate, potentially increasing the risk of sudden cardiac arrest [57]. These models show that the ionic remodelling mechanisms reported in diabetic cardiomyocytes can contribute towards SAN malfunction and therefore contribute to increased arrhythmia risk in diabetic patients.

Action potential models of animal and human ventricular myocytes have been used to investigate the electrical activity of diabetic cells [54, 98–101]. Pandit et. [54] al used a model of the rat ventricular action potential to simulate a diabetic cell by altering the properties and density of potassium and calcium currents and of the sodium–potassium pump. The same action potential model has been modified to replicate dynamics from rat papillary muscles [101]. Ionic current measurements from isolated myocytes of healthy and diabetic rats were then used to find which currents were altered in diabetes. Consequently, the NCX and potassium currents were reduced in the model to show that diabetic cells exhibit APD prolongation [101]. More recently, human models have been applied to investigate the effects of diabetes on the human ventricular action potential [100, 102, 103]. A model for the human ventricular action potential has been used by Fouda et al. [102, 103] to show that high glucose affects the activation of the voltage-gated sodium channels in a concentration-dependent manner, leading to decreased upstroke velocity and prolonged APD. Importantly, they showed that oestradiol can reverse APD prolongation, highlighting the importance of female hormones in sex-specific arrhythmia risk [102]. Ashrafi et al. [100] used the same action potential model in combination with messenger RNA data collected from aortic valve surgery patients with and without diabetes to alter the function of the sodium–potassium pump and SERCA to replicate the action potential of diabetic cells. These alterations caused APD prolongation and early afterdepolarisation in endocardial cells, potentially explaining the increased arrhythmia risk of diabetic patients.

### Calcium handling models

Altered calcium handling has been identified as a factor contributing towards HF progression in diabetes. Therefore, understanding the mechanisms underlying calcium handling in the diabetic cell is important to find new potential treatment targets and prevent HF progression. Das et al. [49, 50] developed a model to simulate calcium handling in cardiomyocytes including L-type calcium channels (LCC), ATP consumption by SERCA, the RyR, the NCX and, importantly, glucose transporter type 4 (GLUT4), the transmembrane protein responsible for glucose uptake in cardiac cells. Diabetes was simulated by decreasing glucose reabsorption, and the model was used to show the importance of GLUT4 in determining SERCA function and therefore calcium handling in the cell. Calcium handling is also affected by amylin, a hormone released by the pancreas and that increases in diabetes. To understand the effect of increased membrane permeability due to amylin combined with SERCA malfunction, Stewart et al. [104] developed a calcium handling model and demonstrated that increased membrane permeability to calcium was able to raise intracellular calcium levels, potentially linking amylin and therefore pancreatic to cardiomyocyte function. A model of calcium fluxes in a rat cardiomyocyte has been used to identify altered calcium handling mechanisms in diabetes [99]. By estimating the model parameters to match intracellular calcium measurements in healthy and diabetic Langendorff-perfused rat hearts, Op Den Bujis et al. [99] were able to show that SERCA activity was reduced in diabetic cells. In a similar study by the same group [105], the authors demonstrated that rat diabetic hearts have a less pronounced calcium and contractility response to beta-adrenergic stimulation compared to normal hearts. Diabetes is also characterised by arterial stiffening [106], which may be partly due to changes in calcium handling in arterial cells. To investigate this, Morotti et al. [98] modified a rat model for arterial myocytes calcium and electrophysiology dynamics to include the effects of glucose. The simulations showed that the LCC were the primary determinant of arterial dynamics in hyperglycaemia conditions, potentially identifying the arterial L-type calcium current as a major contributor to vasoconstriction.

**Fig. 3:**
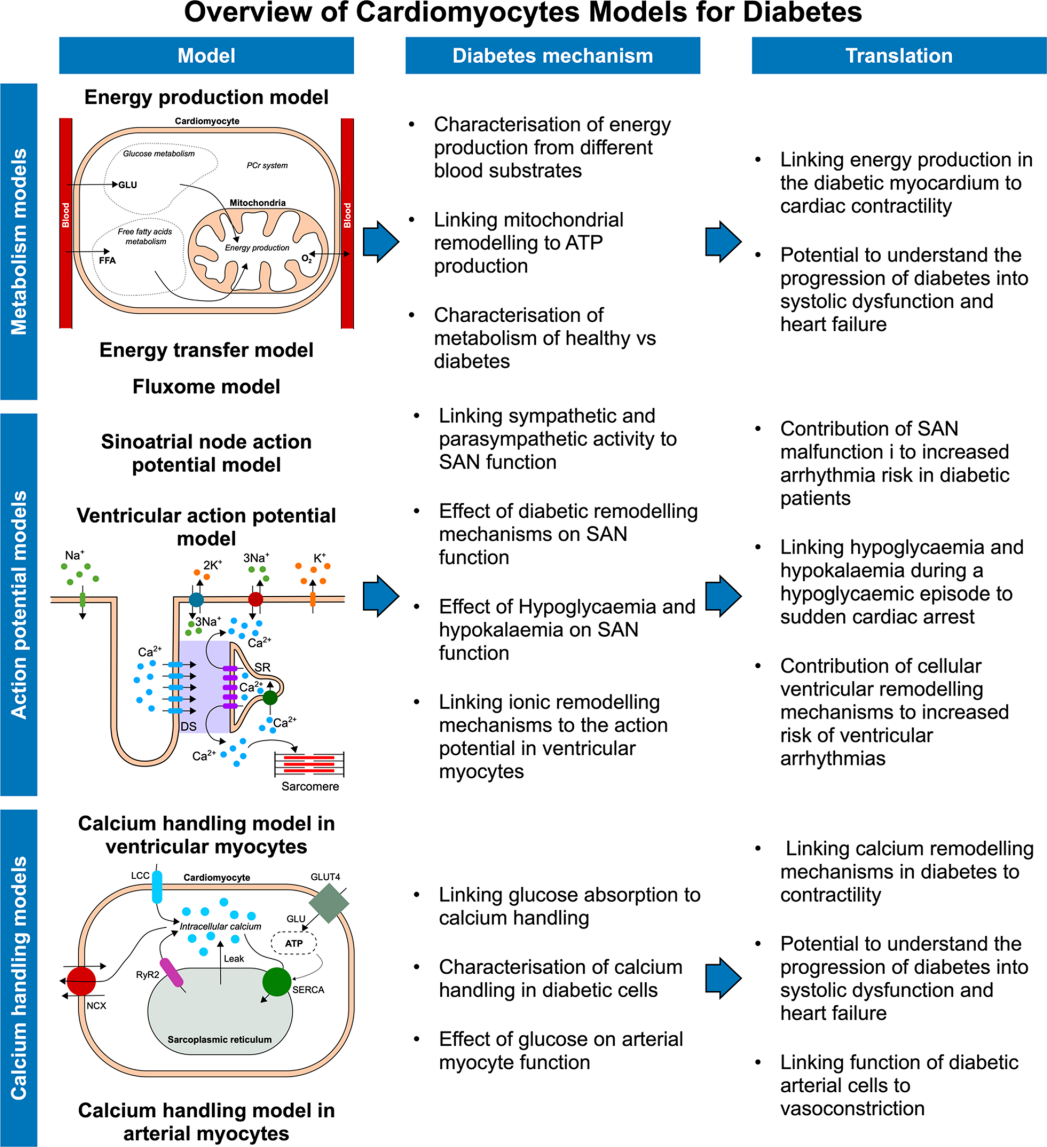
Overview of cardiomyocyte models for diabetes. Models for cellular metabolism in diabetes have been used to understand and characterise energy production in diabetic cells. Animal and human action potential models of the sinoatrial node (SAN) and ventricular myocytes were applied to quantify the effect of sympathetic and parasympathetic activity, diabetes ionic remodelling, hypoglycaemia and hypokalaemia on SAN and ventricular cardiomyocytes electrical activity. Calcium handling models for cardiomyocytes and arterial cells were able to quantify the effect of glucose on calcium dynamics. These models have the potential to improve our understanding of how diabetes cellular remodelling can lead to systolic and diastolic dysfunction, and increased arrhythmia risk. *GLU* glucose, *FFA* free fatty acids, *O*_*2*_ oxygen, *PCr* Phosphocreatine, *Na*^+^ sodium, *K*^+^ potassium, *Ca*^*2*+^ calcium, *SR* sarcoplasmic reticulum, *DS* dyadic space, *LCC* L-type calcium channels, *NCX* sodium-calcium exchanger, *RyR2* ryanodine receptor, *SERCA* sarcoplasmic/endoplasmic reticulum calcium ATPase

Computational studies using animal and human action potential models are an invaluable tool to explain experimental observations on diabetic cells, quantify the effect of diabetes remodelling mechanisms on the action potential, calcium handling and contractility, and bridge the gap between experimental observations on healthy and diabetic animals and clinical practice. However, these models still need to be integrated at the whole heart scale, to quantify the effects of diabetes remodelling on whole-organ function and to improve our understanding of how the diabetic heart degenerates into HF. Figure 3 provides a summary of cardiomyocyte models, their contribution towards understanding cellular dynamics in diabetes and their potential for clinical translation.

## Blood flow models in diabetes

**Fig. 4:**
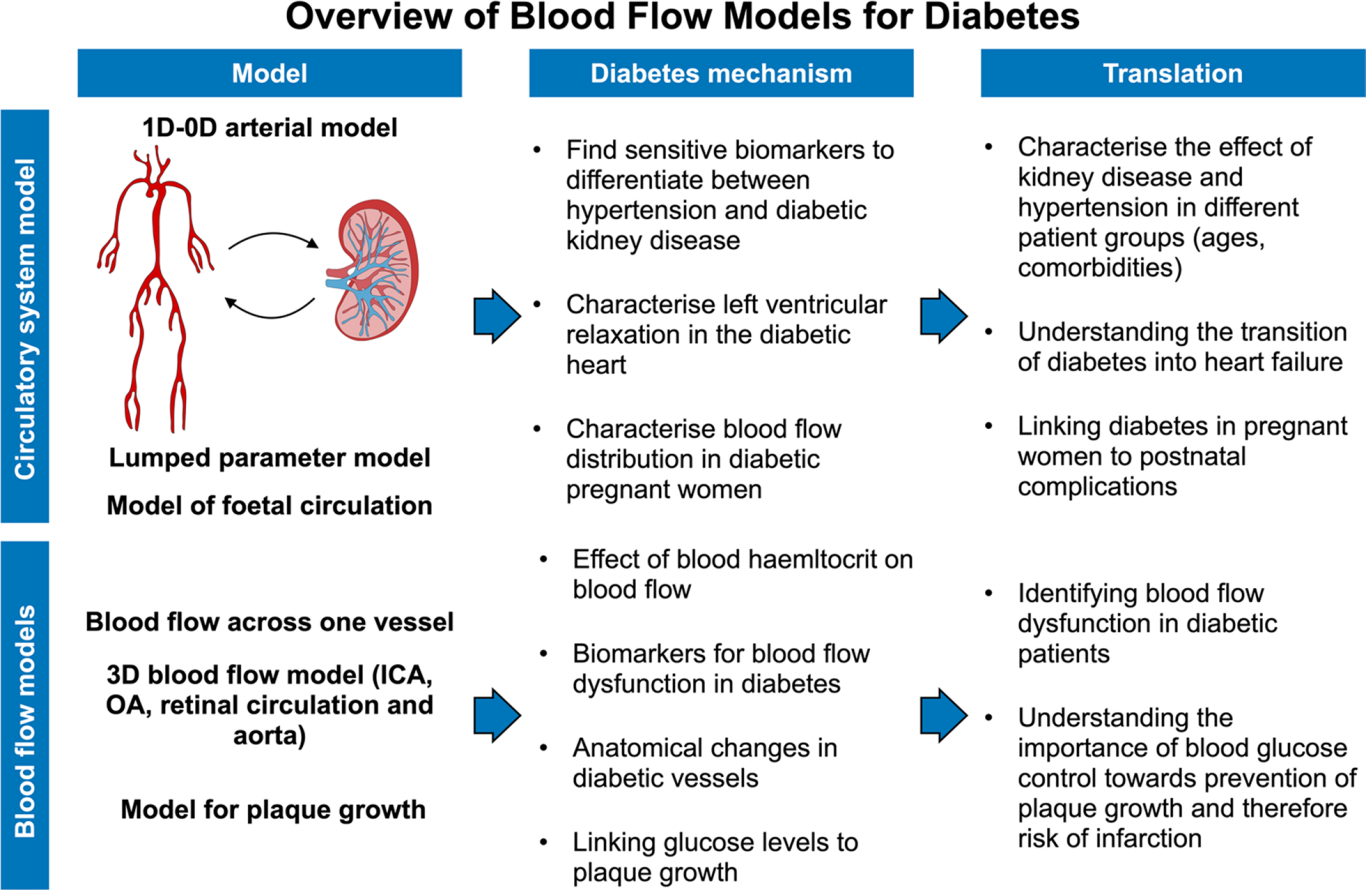
Overview of blood flow models for diabetes. Arterial blood flow models and lumped parameter models have been used to find more sensitive biomarkers to differentiate between different comorbidities in diabetes, and to characterise ventricular relaxation and blood flow distribution in diabetic pregnant women. CFD and local blood flow models of different arteries (*ICA* internal carotid artery, *OA* ophthalmic artery) were used to investigate the effects of diabetes on blood flow across diabetic vessels and quantify the effects of glucose on plaque growth

The metabolic shift caused by increased glucose levels leads to inflammation and endothelial dysfunction, which in turn induces plaque formation and impaired vasodilation of the vascular bed [32]. Hyperglycaemia damages major coronary and systemic arteries and leads to increased arterial stiffness, which can contribute towards the development of hypertension and kidney damage [2]. In addition, hyperglycaemia has direct consequences on renal function, as it increases the glomerular filtration rate and therefore renal arterial pressure, and it initiates inflammation and fibrosis within the kidneys [31]. These mechanisms increase both the afterload and the preload of the heart through increased peripheral resistance and arterial stiffness and through fluid retention and increased circulating blood volume. Understanding how the circulatory system and the kidneys affect cardiac function is vital to identify new treatments for diabetes. This is of particular importance in the context of SGTL2i and GLP1-RAs, directly targeting the kidneys [107] that were also shown to have cardioprotective effects [107–110]. Blood flow computational models offer a mathematical representation of blood flowing through blood vessels and/or different compartments of the circulatory system. Although they constitute simplification of complex blood flow dynamics of the diabetic heart and circulation, blood flow modelling has the potential to link kidney function, the circulatory system, and the heart by incorporating these elements in a coupled framework where the heart and arteries provide blood to the kidneys, and kidney function modulates glucose, sodium and water retention leading to changes in cardiac preload and mean arterial pressure. This can serve as a unique tool to understand how cardiac function adapts in response to systemic changes due to diabetes and anti-diabetic treatment. In Fig. 4, we summarise blood flow models applied to understand the effects of diabetes on blood flow.

### Circulatory system models: linking the heart to the cardiovascular system

Circulatory system models have been used to study the effects of diabetes on the systemic arteries and arterial circulation [111–113]. A one- and zero-dimensional (1D-0D) model of arterial and renal circulation has been applied to simulate blood flow through the arterial tree and the kidneys in patients in different age groups, with or without diabetes or hypertension, by altering the mechanical and geometrical properties of the vessels [112]. This computational study provided a new potential biomarker to differentiate between hypertension and diabetic renal disease. Tunedal et al. [113] employed a lumped parameter model of the left side of the circulatory system in combination with MRI and blood pressure data to generate patient-specific models of controls, patients with or without diabetes, and with or without hypertension. The model parametrisation revealed that only patients with both diabetes and hypertension had impaired LV relaxation and that the interaction between circulatory system remodelling and emergent cardiac function is complex and arises from the combination of different mechanisms. A lumped parameter model of mother and foetal circulation was used in combination with Doppler data from the foetus’s heart to understand the differences between blood flow in diabetic and non-diabetic mothers [111]. The model showed that, in diabetic mothers, the blood flow is redistributed away from the foetus’ brain into the placenta, which might play a role in postnatal complications. Blood flow and circulatory system models are flexible and efficient, and they have been used in a wide range of applications, from renal to foetal circulation. However, these models do not include a detailed representation of the microvasculature, whose properties are altered by diabetes. The microvasculature is typically lumped into one compartment in the simulation framework and therefore represented by resistance, compliance and inductance elements. The endothelial damage of major arteries occurring in diabetes can be represented as altered mechanical properties of the arterial walls (e.g., increased stiffness), but dynamics for plaque formation, growth and its effects on local blood flow cannot be accounted for in these models. Nevertheless, the modularity of circulatory system models allows extending them to include a more complex representation of different compartments of the circulatory system to make them more suitable for different applications.

### Understanding blood flow in diabetic vessels

Diabetes causes the systemic and coronary arteries to become stiffer and narrower, leading to changes in local blood flow which are challenging to measure in-vivo. Branigan et al. [114] used an analytical model for blood flow in a compliant vessel in response to different levels of haematocrit in healthy and diabetic patients and were able to show that the mechanical properties of diabetic vessels were altered compared to normal subjects. More complex, three-dimensional (3D) CFD models provide information about local changes in blood velocity and shear stresses. Ahmed et al. [71] used a 3D anatomical model of a human carotid artery and altered blood viscosity parameters to simulate healthy and diabetic conditions. The model demonstrated that, in diabetes, blood velocity decreased, and wall shear stress and pressure increased, therefore linking blood parameters to the mechanical function of the carotid artery. CFD has also been used to study blood flow in the aorta of children with and without type 1 diabetes [72]. By building patient-specific models, Samyn et al. were able to show that wall shear stress differed between diabetics and controls, while global MRI-derived aortic distensibility was not significantly different. CFD-derived metrics might therefore be more sensitive biomarkers for blood flow dysfunction in diabetic subjects.

The endothelial dysfunction induced by hyperglycaemia increases the risk of stenosis in the major vessels. Understanding the mechanisms and consequences of plaque growth is fundamental to finding a better way of preventing and managing cardiovascular complications in diabetes. A mathematical model of plaque growth in diabetic conditions has been developed by Xie et al. [115–117], accounting for normal and abnormal levels of high- and low-density lipoproteins and blood glucose. The authors used the model to show that, in hyperglycaemia conditions, the plaque persists even at normal cholesterol levels, highlighting the additional risk that high blood glucose poses in terms of plaque formation [117]. Behir et al. [118] used CFD dynamics to simulate 2D blood flow in an idealised cylindrical stenotic vessel in the presence of diabetic haematocrit levels. High haematocrit, typical of diabetic patients, further decreased blood flow through the stenotic vessel, therefore potentially exposing diabetic patients to an increased risk of cardiovascular complications compared to non-diabetic patients.

Diabetic patients are also more likely to develop vision loss due to impaired blood flow to the eye [45]. Shape modelling of the retinal circulation has been used to quantify the link between retinal vascular shape and the progression of diabetic retinopathy [119]. CFD modelling of the carotid and ophthalmic artery, the vessel responsible for blood supply to the eye, has been used to compare geometry and functional parameters of blood flow in diabetic and non-diabetic patients, and with or without acute coronary artery syndrome [48]. Anatomical patient-specific models of the ophthalmic artery were built from head and neck CT angiography images and were used to demonstrate that the diabetic vessels were smaller in diameter. Blood flow simulations showed that patients with both diabetes and acute coronary syndrome had smaller velocity and higher pressure than controls, and that mass flow ratio between the ophthalmic and the carotid artery correlated with cardiac biomarkers (e.g., NT-proBNP), therefore potentially linking peripheral blood flow to cardiac function. Finally, CFD models of retinal microaneurysms have been applied to compute local shape and blood flow biomarkers to determine which microaneurysms are more likely to clot and therefore lead to diabetic retinopathy [120]. CFD models can be used to improve our understanding of blood supply to the peripheral circulation in diabetic and non-diabetic patients and to understand the link between diabetes and complications following damage to both big and small vessels.

Fluid dynamics modelling provides a non-invasive platform to investigate how blood flow through different types of vessels changes due to anatomical, rheological, and mechanical properties. In particular, 3D CFD offers detailed information about the anatomical remodelling of blood vessels due to diabetes, and about local shear stresses and pressure in the vessels, potentially providing new and more sensitive biomarkers to identify diabetic patients who are more at risk of developing macrovascular and microvascular complications. However, they only provide indirect information about peripheral and myocardial perfusion, as they lack a representation of microvasculature dynamics, which are also altered by diabetes and lead to reduced peripheral circulation and multi-organ dysfunction. Combining 3D CFD with perfusion models has the potential to improve our understanding of the perfusion of the peripheral circulation and the myocardium in diabetes.

## In-silico trials: understanding the effect of anti-diabetic treatment on the heart

CVOTs investigate the cardiovascular effects of anti-diabetic drugs. More recently, SGLT2i and GLP1-RAs have been shown to provide cardioprotective mechanisms, but the underlying mechanisms remain unclear. Understanding how these drugs work on different patient groups will contribute towards better patient selection and outcome. In this context, cardiac computational models and in-silico trials can help by simulating drug mechanisms of different virtual cohorts of patients, comparing response, and testing different hypothesised mechanisms of action on the heart. Furthermore, with the increasing availability of anti-diabetic drugs with varying levels of positive effects, cardiac computational models could be used to assist in deciding which drugs to prioritise, therefore leading to the optimal treatment course for the patient.

**Fig. 5:**
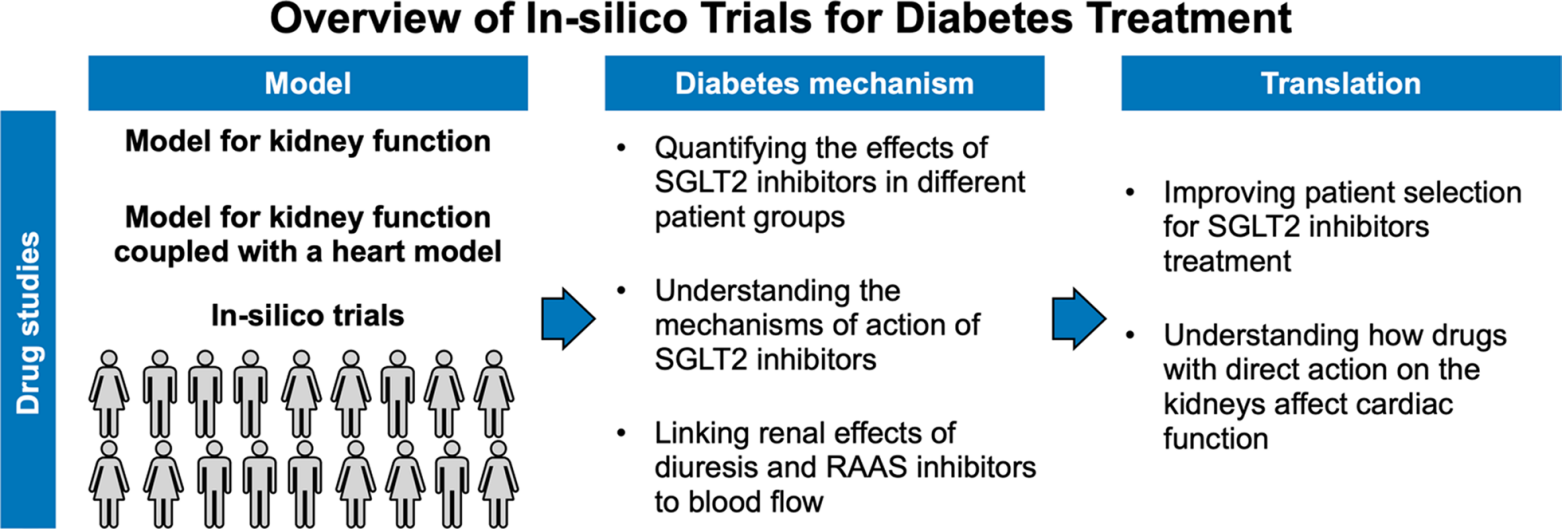
In-silico trials for diabetes treatment. Lumped parameter models of different complexities were run to perform in-silico trials to understand the effects of SGLT2i and other drugs with direct effects on the kidneys on the heart

To date, cardiac computational models have been used to investigate the cardiac effects of SGLT2i, showcasing their potential to improve our understanding of the mechanisms of action of anti-diabetic drugs (Fig. 5). Models for water and sodium clearance by the kidneys alone or coupled with models for the circulatory system and heart have been used to investigate indirect and direct effects of SGLT2i on the heart [78–81, 121]. Hallow et al. [121] used a mathematical model to link diuresis and sodium clearance to blood and interstitial fluid in response to dapagliflozin. This model was then integrated into a more complex framework for kidney circulation in combination with clinical data for renal function collected from controls undergoing dapagliflozin treatment [79]. Simulating the effects of dapagliflozin on the kidney showed that inhibition of the sodium-hydrogen exchanger (NHE3) in the kidneys improves renal function and reduces interstitial fluid and blood volume. This could be one of the modes of action of dapagliflozin. Yu et al. further extended this simulation framework to couple it with a model for cardiac remodelling and contractility [78]. The model was then used to build a virtual population of type 2 diabetes patients with normal cardiac function, constrained using the blood pressure and kidney function data from the DAPACARD trial [122]. The virtual cohort was then modified to simulate HF with reduced ejection fraction (HFrEF). The study demonstrated that while dapagliflozin does not affect myocardial strain and cardiac efficiency in patients with normal cardiac function, improvements in systolic function can be achieved in patients with reduced ejection fraction at baseline. Following a similar approach in combination with data from the DAPA-HF trial [110], the same group showed that the renal effects of dapagliflozin lead to decreased end-diastolic volume and pressure in HF patients [80]. Yu et al. [81] used a further extended version of the model including more detailed regulatory mechanisms for kidney function and combined it with clinical data from the RADAR clinical trial [123] to investigate the effects of renin–angiotensin–aldosterone system inhibitors and diuretics on blood pressure. These studies constitute examples of how *in-silico clinical trials* can contribute towards our understanding of drug mechanisms of action in different cohorts of patients, to identify patient groups that are more likely to benefit from a specific treatment.

## Discussion

In this review, we describe the cardiac computational models that have been applied in the context of diabetes. Models of cardiomyocyte metabolism have been used to characterise energy production in diabetic cells and to quantify the effects of mitochondrial structural and anatomical remodelling. Ionic remodelling mechanisms in diabetes and their effects on the action potential and calcium handling have been investigated with animal and human action potential, calcium handling and contractility models, providing a potential link between remodelling of diabetic cells and arrhythmia risk, and impaired cell contractility and relaxation. Blood flow modelling has been applied to study how anatomical, mechanical, and blood properties in diabetes affect blood flow through diabetic arteries. Finally, circulatory system models of varying complexities have been employed to link cardiac and kidney function and to perform in-silico trials to better understand the mechanism of action of anti-diabetic agents on different patient populations. Despite the contribution of these models towards our understanding of the diabetic heart and its treatment, there are many opportunities for the development of more sophisticated models extending to patients with different comorbidities.

### The potential of multi-scale and perfusion models for diabetes applications

Hyperglycaemia initiates a plethora of remodelling mechanisms, from metabolic and mitochondrial dysfunction, ionic remodelling mechanisms, and altered calcium handling, to hypertrophy, myocardial stiffening, and microvasculature and macrovasculature complications [15, 27, 31, 38, 39, 41]. While the models discussed in this review provide valuable insight into the effects of diabetes and anti-diabetic drugs on the heart and cover a wide spectrum of physics (metabolism, electrophysiology, mechanics, and flow), they mostly focus on one scale in isolation (cell, arteries, or circulation). Incorporating the multi-scale remodelling mechanisms of diabetes into a multi-scale, whole-heart electromechanics framework would allow us to systematically quantify the contribution of each mechanism towards cardiac function deterioration. Over the past decade, several frameworks for whole-heart, multi-scale electromechanics simulations have been developed [60, 124–127]. This more complex type of model allows single-cell dynamics to be quantitatively linked to whole-organ cardiac function. In a study from our group [60], we used a multi-scale, whole-heart electromechanics framework to perform a sensitivity analysis, where the model parameters representing different scales of cardiac function (from ionic currents and contractility to the circulatory system) were varied to quantify their effect on clinically relevant biomarkers (e.g., end-diastolic volume, end-systolic volume, peak systolic pressure). This resulted in an interaction map between a wide range of model parameters and clinically relevant indices of cardiac performance, showing the potential of this type of framework to link processes at different scales leading up to global cardiac contractile function, which is not possible to do through clinical data alone. Whole-heart electromechanics frameworks developed so far however do not account for cardiomyocyte metabolism. Integrating a highly detailed model for cell metabolism and mitochondrial function following the Cortassa et al. [92, 94] or even a simpler approach to include the effect of glucose transport on ATP production as in Das et al. [49, 50] would allow the model to be extended further to simulate the effects of altered metabolism, central for the diabetic heart. Given their complexity and wide range of dynamics that they can simulate, whole heart electromechanics models have the potential to provide a non-invasive platform to understand the diabetic heart, and to help improve the treatment, management, and stratification of diabetic patients in terms of CVD risk.

Macro and microvascular dysfunction are a hallmark of diabetes and can lead to cardiac hypertrophy and poor cardiac perfusion [39]. However, the mechanisms leading to poor perfusion in the diabetic heart remain only partially characterised. Perfusion models have the potential to improve our understanding of myocardial blood supply in the healthy and diabetic heart. Computational frameworks have been developed to simulate blood flow across the coronary tree, spanning from the major coronary vessels down to the coronary microcirculation [73]. However, this type of model has not been applied to investigate the diabetic heart. A myocardial perfusion model could be personalised to simulate blood flow distribution in diabetic and non-diabetic patients to determine which properties of the coronary tree are most likely to lead to poor myocardial perfusion and impaired cardiac function, therefore linking different scales of blood flow in the diabetic heart.

### Understanding sex differences in the diabetic heart

Data from the Framingham Heart Study showed that diabetic women have a higher risk of developing cardiovascular complications compared to diabetic men [128]. The diabetic female heart was also shown to remodel more severely due to glucose intolerance [129], and to have more significantly reduced cardiac systolic function [130]. However, the reasons behind these differences in the relation between diabetes and the heart have yet to be elucidated [131]. In this context, sex-specific cardiac computational models can help understand the differences between the male and female diabetic hearts, and to understand whether response to anti-diabetic treatment differs between sexes. Sex steroid hormones (mainly oestrogen, progesterone, and testosterone) affect ionic currents in cardiomyocytes and action potential models of the human ventricular myocyte have been used to quantify their effect on the action potential and drug cardiotoxicity [132, 133]. These have been subsequently incorporated into multi-scale electrophysiology models to quantify the effect of sex steroid hormones and drug-induced arrhythmias at the whole organ level [134, 135]. Electrophysiology models for LV activation have also been employed to understand sex-specific response to cardiac resynchronisation, which was attributed to smaller heart size in women compared to men [136]. Size differences between sexes do not only concern the heart, but they have been found in the great vessels as well [137]. Gao et al. used CFD simulations to demonstrate that these anatomical differences translate into an increased risk of aortic aneurysm rupture, therefore linking anatomical and functional sex differences [138]. Finally, cardiac computational models have been used to study how a woman’s cardiovascular system adapts in pregnancy due to hormones and increased demand for blood flow to the placenta and growing baby [139]. Apart from the study from Fouda et al. [102], quantifying the effect of oestradiol on APD, and the lumped parameter model developed by Kulkarni et al. [111], used to quantify differences in foetal blood flow between diabetic and non-diabetic pregnant women, sex-specific models of the heart and cardiovascular system have yet to be applied to diabetes. Applying cardiac computational models to understand the effects of diabetes on the male and female heart could potentially explain the increased risk of CVD complications observed in diabetic women compared to diabetic men. Further, cardiovascular response to anti-diabetic treatment in women and men could be quantified with a computational framework similar to the one developed by Yu et al. [78, 80], to investigate whether the differences observed between the male and female diabetic heart translate into a difference in response to anti-diabetic treatment. This is of fundamental importance, given that CVOTs systematically enrol more men than women [107–110, 128] despite the evidence that diabetic women have worse prognosis when it comes to cardiovascular complications [128].

### The interaction between diabetes and obesity

Diabetic patients, particularly those with type 2 diabetes, often suffer from obesity. Diabetes and obesity independently increase the risk of CVD and, in combination, they are collectively associated with higher cardiovascular mortality [140]. However, because 90% of patients with type 2 diabetes are overweight, it is difficult to distinguish the relative contribution of each towards CVD. Marciniak et al. [141] built a statistical shape model of the LV from over 2000 MRI scans of children to investigate differences between obese and non-obese children. Unique anatomical features were linked to childhood obesity, providing biomarkers for LV shape for risk stratification. However, the shape was not linked to cardiac function. Including anatomical variability as a function of the body mass index or body surface area in a multi-scale whole heart model would provide a link between shape and function [61]. This has the potential to identify patients at higher risk of CVD that might benefit from lifestyle modification through more aggressive weight loss strategies, to reduce the additional burden caused by obesity on the diabetic heart.

The interaction between diabetes and obesity causes uncertainty on how to best treat patients who suffer from both. Recently, GLP1-RAs, a new anti-diabetic drug class causing insulin secretion through pancreatic cell stimulation, have been shown to prevent the development of diabetes in pre-diabetic subjects, to lower Hb1Ac and, importantly, to reduce gastric emptying and therefore encourage weight loss [142]. In addition, they were shown to decrease blood pressure and improve LV myocardial strain [142], although the precise mechanisms through which this happens remain unclear. While it has been shown that the reduced CVD risk following GLP1-RA treatment is independent of glycaemic control [143], the relation between weight loss and LV function and cardiovascular mortality remains poorly understood. Bariatric surgery constitutes an alternative to drug treatment when it comes to obesity, and it has been shown to reduce LV mass and increase LV global longitudinal strain without significant changes in LV end-diastolic and end-systolic volumes [142]. The long-term effects of bariatric surgery on cardiac function in diabetic patients remain unknown. Cardiac computational modelling studies focusing on diabetes treatment published so far have focused on SGLT2i. A cardiac computational model able to replicate the separate effects of weight loss and reduced blood glucose due to GLP1-RAs or surgical intervention has the potential to enhance our understanding of the interaction between these factors, and to improve the care of diabetic and/or overweight patients.

### In-silico trials: a tool to accelerate cardiovascular outcome trials

The studies by Hallow et al. [79] and Yu et al. [78, 80, 81] discussed above constitute examples of in-silico trials applied to understand response to SGLT2i in different patient populations (healthy or HF). Because they are mainly focused on the kidneys, these frameworks lack a detailed, multi-scale representation of the heart. Therefore, the information they provide about the heart remains limited to whole-organ features (e.g., pressure and volumes). Following a similar approach, in-silico trials for diabetes could be carried out with a framework including a more complex representation of the heart, where not only the circulatory system was included, but also ionic, calcium handling and contractility processes. SGLT2i directly target the kidneys, but off-target mechanisms explaining their cardioprotection through NHE inhibition in the heart [144, 145], reduced late sodium currents [146], or reduced calcium/calmodulin-dependent kinase function [147] have been hypothesised and demonstrated by animal experiments. However, these mechanisms of action, particularly the effect of SGLT2i on the NHE in the heart, remain debated [148, 149]. Including these hypothesised mechanisms of action in an in-silico trial with a model accounting for the multi-scale function of the heart has the potential to provide clarity about how SGLT2i provide cardioprotection.

Performing in-silico trials on different patient populations, again following the approach by Yu et al. [78, 80], would provide information about which patients would benefit the most from SGLT2i therapy. This is of fundamental importance for diabetic patients with additional cardiac conditions, as diabetes worsens the symptoms and prognosis in patients with existing CVD. In patients with non-ischemic dilated cardiomyopathy, diabetes increases LV mass, reduces LV function, and worsens clinical outcomes [150, 151]. On the other hand, hypertrophic cardiomyopathy patients with diabetes had similar LV function to non-diabetics but had enlarged atria, higher incidence of atrial fibrillation, pulmonary hypertension, increased fibrosis burden, decreased left atrial ejection, worse myocardial energetics, and lower long-term survival [152, 153]. Diastolic dysfunction is one of the hallmarks of the diabetic heart, with about 50% of diabetic patients with HF presenting with HF with preserved ejection fraction (HFpEF) [154]. Diabetic HFpEF patients were shown to have a worse prognosis than their non-diabetic counterparts, but they are also younger and more obese [155]. This shows that diabetes interacts with existing cardiac conditions (e.g., dilated cardiomyopathy, hypertrophic cardiomyopathy, HFpEF), worsening cardiovascular outcomes. Furthermore, the DAPA-HF trial [110] demonstrated that the cardiac benefits provided by SGLT2i were similar between patients with and without type 2 diabetes, indicating these cardioprotective mechanisms might, at least partly, not be derived from treating the diabetic heart. Performing in-silico trials on different populations of patients with and without diabetes would provide a unique opportunity to non-invasively test the effect of anti-diabetic treatment on different patient sub-groups, potentially assisting patient selection and enrolment in CVOTs and ultimately may aid in developing more bespoke treatment algorithms among diabetic patients with different CVD.

Anti-diabetic medications have been under scrutiny when it comes to their consequences on CVD risk. Metformin, the most prescribed glucose-lowering agent, was first introduced in 1957 and was shown to improve cardiovascular outcomes by the United Kingdom prospective diabetes study (UKPDS) [156]. However, these effects were evident 6 years after treatment and, because metformin was introduced into diabetes care 60 years ago, it did not benefit from being tested in rigorous CVOTs [156]. This makes it difficult to compare the cardiovascular effects of metformin and more recent anti-diabetic drug classes. Other medications, like glitazones and dipeptidyl peptidase-4 (DPP-4) inhibitors, have been tested for their effects on CVD risk. Glitazones cause fluid retention, potentially leading to increased HF risk and hospitalisation [157, 158]. However, they were also shown to reduce the risk of events related to atherosclerosis and atrial fibrillation [157]. Therefore, glitazones might still be beneficial, especially when used in combination with SGLT2i to counteract fluid retention [157] The SAVOR-TIMI 53 trial demonstrated that the DPP-4 inhibitor saxagliptin increased the risk of HF hospitalisation [159], therefore raising concerns about the use of this drug class in patients HF or pre-existing LV dysfunction [160]. The SAVOR-TIMI 53 alone enrolled 16,492 patients with a median follow-up of 2.1 years. Although CVOTs are necessary to ensure patient safety, they require a lot of time and resources. A model able to capture the mechanisms of action of interest to simulate the effects of different anti-diabetic treatments on the heart has the potential to assist the design of CVOTs and to optimise targeted patient enrolment. Furthermore, with the increased availability of anti-diabetic treatments with varying levels of cardioprotection, cardiac computational models have the potential to help design the treatment course of a patient, including which drug classes to prioritise. This holds future promise to optimise and personalise patient treatment, accelerate the translation of new anti-diabetic treatments to the market, and ultimately improve the care of diabetic patients.

### Future directions: cardiac digital twins and in-silico trials

One of the unmet clinical challenges of diabetes is how to manage diabetic patients with different CVD risk. Given the increasing number of available anti-diabetic therapies, it remains unclear which drug to give first and to which patients, leading to sub-optimal treatment strategies. In this context, precision medicine, where the treatment is tailored to a specific patient, can help provide the best treatment faster for every diabetic patient. A *digital twin* able to represent the patient’s heart and circulatory system has the potential to predict response to different available therapies and to help general practitioners and cardiologists select the optimal drug at the optimal time.

A digital twin is a virtual replica of a physical entity, system, or process, designed to replicate its real counterpart accurately. It spans the lifetime of the physical entity, it is updated over time to reflect real-world changes, and it assists in monitoring, analysing, and predicting the response of the physical entity to different conditions or stimuli. In healthcare, a digital twin of a patient needs to be updated over time with the available clinical data collected, for example, during a hospital visit or a doctor appointment and could help optimise the timing of a check-up, anticipating cardiovascular events or hospitalisation, and predicting response to treatment. Before the digital twin concept can be realised in the context of medicine, several challenges concerning expensive model personalisation procedures and data integration remain to be addressed [161].

Digital twins can be used in combination with in-silico trials to mirror a CVOT. Given the cost and time required by a CVOT, having a corresponding in-silico trial to run beforehand would optimise patient recruitment by providing quantitative data for power calculations, therefore making the CVOT more efficient. In-silico trials have also the potential to improve patient representation in CVOTs. Despite the known increased risk of diabetic women compared to diabetic men, CVOTs so far have systematically enrolled more men than women. With in-silico trials, we would be able to increase the number of women in the virtual population by either building more female cardiac digital twins or by generating more virtual patients using population statistics. Following a similar approach, in-silico trials have the potential to reduce the race/ethnic and socioeconomic disparities in diabetes and in diabetes treatment. Race/ethnic minorities have higher prevalence of diabetes alone and in combination with CVD and higher mortality rates due to diabetes [162, 163]. Given this increased risk, future research needs to focus on understanding the reasons behind these differences, which remain partially unexplained. In addition to designing in-silico trials with balanced virtual populations, this technology could be used to quantify differences in treatment response between sexes and ethnicities. This has the potential to provide more equitable care to the increasing number of diabetic patients around the world.

### Limitations of cardiac computational models

Despite their potential, computational models of the heart and cardiovascular system have limitations. The diabetic heart is characterised by a plethora of multi-scale and multi-factorial remodelling mechanisms, from myocyte metabolism, electrophysiology and calcium handling to inflammation leading to dysfunction of the micro- and macro-vasculature and consequently to multi-organ dysfunction. Cardiac computational models for diabetes constitute a simplification of these mechanisms, leading to compromises. The models only represent a pre-defined subset of biological pathways, and this may limit their ability to identify novel disease or physiological mechanisms. However, the pathways that are represented are explicitly known, they can all relate to the mechanisms of interest, and the impact of ambiguity in the biology or model incompleteness can be estimated using uncertainty quantification. Further, models allow all extensive measurements to be made simultaneously across multiple scales (from cell to whole organ and circulation), and extensive experimental designs can be performed at negligible cost in time without risk to human or cost of animal lives. This contrasts with an experimental animal model, where the complexity is present, but it may not always be relevant to human physiology. Further, only a limited number of measurements can be made, these may need to be made in separate experiments or settings, and only a limited number of experiments can be performed.

There is no universally accepted gold standard for validation and comparison. This makes it challenging to assess the accuracy, reliability, and generalisability of cardiac computational models. The current lack of regulation of digital health technologies, including cardiac computational modelling, limits their application in practice, as it raises concerns about patient safety and ethics [82]. This contrasts with animal research, where 10–30% of experiments are expected to be unreproducible [164] and the subtilities in experimental protocols are not always meticulously collected. The FDA has released guidance to assess the credibility of cardiac computational modelling and simulations for medical device regulatory submission [165] and, alongside the growing adoption of open-source tools and standards, this will only increase the reproducibility of computational cardiology research.

Cardiac computational models rely on the availability of high-quality datasets (e.g., imaging, invasive recordings) to be built and validated. However, clinical datasets are often imbalanced, leaving women and ethnic minorities under-represented. This challenge is not unique to cardiac computational models and is an issue for clinical trials and the development of artificial intelligence algorithms. The increased awareness of this issue and the growing availability of public databases will hopefully, over time, address this issue.

Models of the heart and cardiovascular system constitute a promising, innovative technology. However, concerns about model bias, fair and secure access to balanced datasets, and lack of standardisation need to be addressed to accelerate their wider adoption into clinical practice.

## Conclusion

Diabetes is a growing pandemic causing an increased risk of CVD. The multi-scale remodelling mechanisms of the diabetic heart, triggered by raised blood glucose levels, and the complex interaction between the heart and the circulatory system make it difficult to tackle and prevent diabetes progression into HF. Cardiac computational models, a fast-growing technology, have been used to investigate different aspects of the diabetic heart, from cardiomyocyte metabolism, electrical excitation, and contractility, up to the whole circulatory system level and flow through diabetic arteries. While these models have contributed towards our understanding of the diabetic heart and the effect of anti-diabetic treatment, there remains potential for more ambitious applications with multi-scale and multi-physics whole-heart models and multi-scale blood flow models, to integrate all these multi-scale remodelling mechanisms into a unique physics-constrained framework. Realising this potential may allow us to quantify the contribution of each remodelling mechanism of the diabetic heart towards cardiac function deterioration, potentially offering new drug targets for anti-diabetic treatment in specific patient groups and ultimately improving diabetes patient care at a lower cost.

## Supplementary Information

Supplementary material 1.

Supplementary material 2.

## Data Availability

No datasets were generated or analysed during the current study.

## Abbreviation

0D: Zero-dimensional
1D: One-dimensional
2D: Two-dimensional
3D: Three-dimensional
AGEs: Advanced glycation end-products
APD: Action potential duration
ATP: Adenosine triphosphate
Ca^2^^+^: Calcium
CFD: Computational fluid dynamics
CT: Computed tomography
CVD: Cardiovascular disease
CVOT: Cardiovascular outcome trial
DPP-4: Dipeptidyl peptidase-4
DS: Dyadic space (space between the sarcolemma within the T-tubules and the sarcoplasmic reticulum in a cardiomyocyte)
ECG: Electrocardiogram
FFA: Free fatty acids
GLP1-RA: Glucagon-like peptide-1 receptor agonists
GLU: Glucose
GLUT4: Glucose transporter type 4
HF: Heart failure
HFpEF: Heart failure with preserved ejection fraction
HFrEF: Heart failure with reduced ejection fraction
ICA: Internal carotid artery
K^+^: Potassium
LCC: L-type calcium channel
LV: Left ventricle
MRI: Magnetic resonance imaging
Na^+^: Sodium
NCX: Sodium-calcium exchanger
NHE: Sodium-hydrogen exchanger
O_2_: Oxygen
OA: Ophthalmic artery
PCr: Phosphocreatine
PCr/ATP: Phosphocreatine to adenosine triphosphate ratio
ROS: Reactive oxygen species
RyR: Ryanodine receptor
SAN: Sinoatrial node
SERCA: Sarcoplasmic/endoplasmic reticulum calcium ATPase
SGLT2i: Sodium-glucose transport protein 2 inhibitors
SR: Sarcoplasmic reticulum
UKPDS: United Kingdom prospective diabetes study
