# Supplementary material for: Cardiac digital twins: a tool to investigate the function and treatment of the diabetic heart
Source: Cardiovasc Diabetol. 2025 Jul 18;24:293. doi: 10.1186/s12933-025-02839-w (PMC12275252; doi:10.1186/s12933-025-02839-w)
Supplement: Supplementary file 1 — Supplementary material 1. [file 12933_2025_2839_MOESM1_ESM.docx]

**Supplement: Primer on Multi-Scale Cardiac Modelling**

To complement our review and provide readers new to computational modelling with a general background, in this supplement we provide a brief introduction to the different types of computational models of the heart and circulatory system, what type of experimental and/or clinical data can be used to build or validate them, and how they can be coupled together to create multi-scale and/or multi-physics computational framework. This Supplement does not aim at providing a complete review of existing cardiac models. Rather, it aims at providing a concise overview to provide clarity and context for the models described in the main manuscript. Fig. 1 shows a summary of the models we will describe below.

We have divided computational models of the heart into three classes:

1. ***Cell models*** (Fig. 1, top), modelling the function of a single myocyte
2. ***Multi-scale models*** (Fig. 1, middle), modelling the function of the whole heart at the tissue level while incorporating cell function
3. ***Flow models*** (Fig. 1, bottom), modelling cardiac perfusion, blood flow through the vessels or the whole circulatory system.

In the sections below, we refer to *model parametrisation* or *calibration* as the procedure where the values of the model parameters (or constants) are estimated to match target experimental and/or clinical data. On the other hand, *model validation* refers to the process of checking that the model predictions or results agree with unseen experimental and/or clinical data that were not used during the parametrisation procedure. Finally, the term *boundary conditions* define the rules according to which a system behaves at its edges or boundaries (for example, the epicardial surface of the heart).


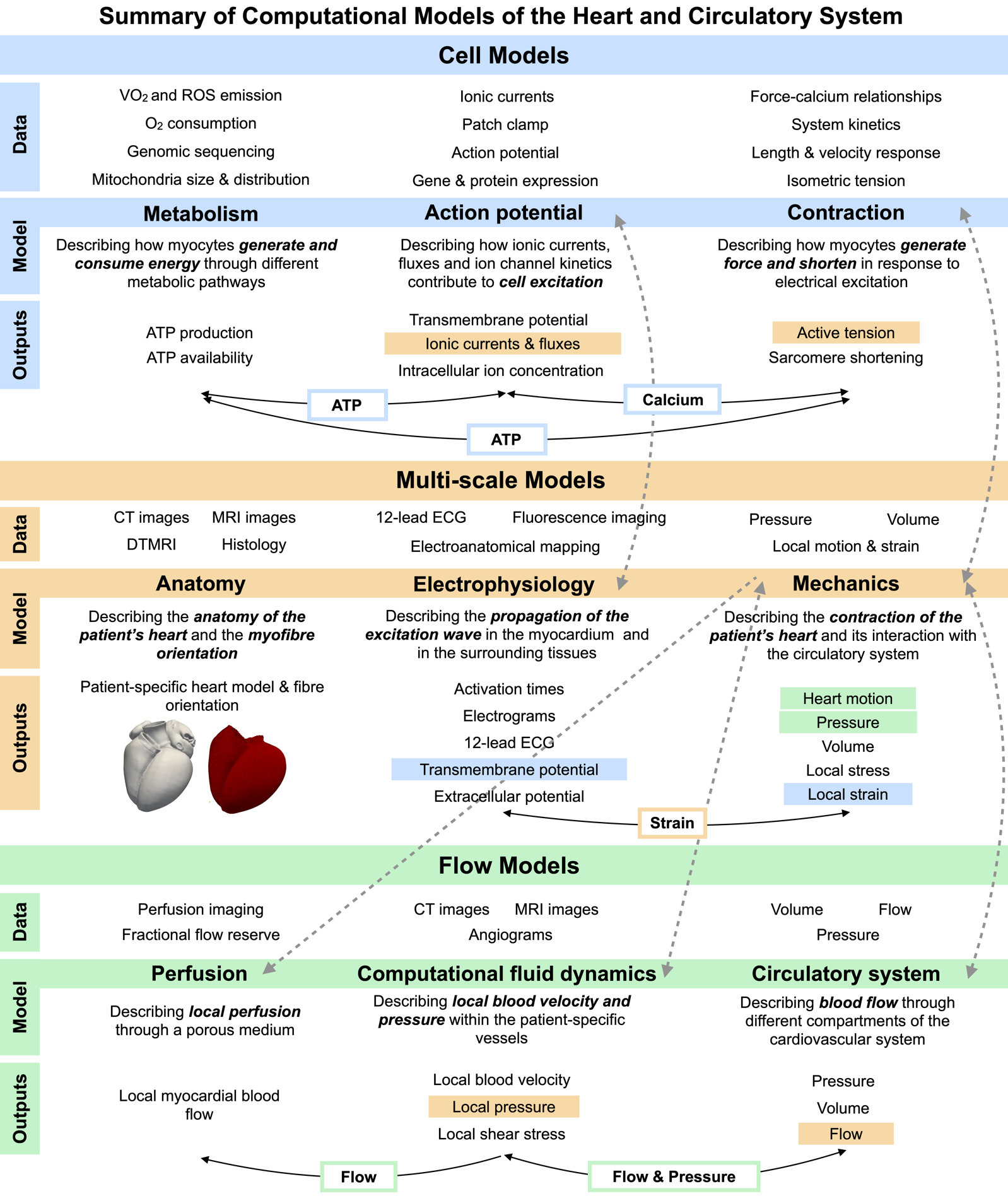


**Figure 1 Summary of computational models of the heart and circulatory system.** For each model class (cell models, multi-scale models and flow models), we summarise the experimental and/or clinical data used to parametrise and validate the models, a short description of the model type and purpose, and the model outputs. The arrows indicate how models can be coupled together. The variables on top of the black arrows indicate the variables through which different model types interact with each other. The gray dashed arrows show how different model classes can be coupled together and the highlighted outputs show how these models interact. For example, action potential models (cell class) provide the ionic currents to the electrophysiology model (multi-scale class), while the latter provides the transmembrane potential back to the cell model. Abbreviations: VO_2_ = respiration rate; ROS = reactive oxygen species; O_2_ = oxygen; ATP = adenosine triphosphate; CT = computed tomography; MRI = magnetic resonance imaging; DTMRI = diffusion tensor MRI; ECG = electrocardiogram.

# 1 Cell models

Cell models can simulate different aspects of the function of a myocyte. Here, we will focus on models for metabolism, electrical excitation, and mechanical contraction. Models for myocyte metabolism represent how cardiac cells produce and consume energy through different metabolic pathways (mainly glycolysis and free fatty acids metabolism). These models can describe cardiac metabolism as a network of biochemical reactions or as a system of equations to compute the products of such metabolic reactions. The first type of models, called *stoichiometric models*, take in input genomic data in the form of a matrix (or table) where the rows are the metabolites, and the columns are the reactions^1^. The reaction fluxes, that is the rate of metabolites used through a specific reaction, are then computed by applying mass conservation to provide information about which metabolic pathways are more active given changes in substrate (e.g., glucose or free fatty acids) availability^2^. The second type of models, generally referred to as *kinetic models*, use a set of equations to describe the rate of different biochemical reactions aimed at energy production^3^. Metabolic models can be calibrated or validated against data collected from isolated mitochondria for O_2_ consumption and respiration rates (VO_2_)^4^. More complex models can also account for cell structure by using data from electron microscopy for mitochondria size and distribution, to incorporate how these geometry characteristics affect energy availability and distribution within the cell^5,6^. In summary, models for myocyte metabolism provide ATP (energy) consumption and availability, which are fundamental for the cell to electrically excite and contract.

Myocyte electrical excitation can be modelled with *action potential models* by simulating the movement of different ion species (e.g., sodium, potassium, and calcium) across the cell membrane or from one cellular compartment to another (e.g., from the cytosol to the sarcoplasmic reticulum). These models typically employ a Hodgkin-Huxley formulation to represent different ionic currents. In this formulation, the current through a voltage-gated ion channel depends on the transmembrane voltage, maximum ion channel conductance and different voltage-dependent gating variables. Owing to the complexity of the electrical activity of a cardiomyocyte, action potential models can have hundreds of parameters. To find the value of the parameters, these models can use data collected from isolated human or mammal myocytes (ion currents, action potential, intracellular ion concentrations). Since conductance parameters indirectly represent the density of different ion channels (e.g., sodium channels, L-type calcium channels), these models can also incorporate gene expression data or proteomics data by scaling the maximum conductance of the channels of interest up or down to capture over or under-expression to represent subject-to-subject variability or a diseased state. Over the past decades, action potential models have been created to represent different cell types (ventricles^7^, atrium^8^, sinoatrial node^9^) and species (rabbit^10^, human^7,11^ and rat^12^). These models provide the transmembrane potential over time and the variation of the intracellular concentration of key ion species, like calcium, which are fundamental for cardiac contraction.

Models for myocyte contraction describe how the cells generate force in response to an electrical stimulus. *Biophysical models*^13–15^ use a set of equations to represent the biochemical reactions leading to cardiac contraction (cross-bridge cycle), from the calcium ions binding to troponin C on the thin filament to detailed representations of cross-bridge mechanics. These models can also account for length (Frank-Starling mechanism) and velocity dependence of contraction. *Phenomenological models*^16^ provide a more simplified approach, where empirical relationships are used to represent active tension generation in response to electrical excitation. Contraction models can be parametrised using experimental data collected from isolated myocytes, trabeculae or tissue strips providing isometric tension generation, force-calcium relationships, or length and velocity dependence response. These models then compute active tension generation and, if coupled with a model for passive mechanics, sarcomere shortening^13^.

The models described above represent one aspect of myocyte function (metabolism, electrical excitation, or contraction), but they can be coupled together to provide a more complete picture of cell function. An action potential model and a model for cell contraction can be coupled together through the calcium transient to represent the electromechanical function of the cell. Action potential models include ion transport through ion pumps (e.g., sarcoplasmic/endoplasmic reticulum calcium-ATPase), which consume ATP to carry out their function. Similarly, ATP is needed during the cross-bridge cycle. Because both the electrical and mechanical activity of the cell require energy, ATP availability and consumption can be used as a coupling variable between a model of myocyte metabolism and an action potential and contraction model^17^. The coupled model can then be used to provide a complete representation of how metabolic changes affect the electrical activity and the contractility of the cell.

# 2 Multi-scale models

Multi-scale models of the heart aim at representing how whole-organ function changes as a function of cell mechanisms (Fig. 1, middle). These models require a three-dimensional (3D) representation of the patient’s heart. Typically, echocardiography, computed tomography (CT) or magnetic resonance images (MRI) are segmented to extract the anatomical structures of interest. The segmentation is then post-processed, and a computational mesh is created and used to run simulations^18^. The model also needs to incorporate the local myofibre orientation within the tissue, fundamental to translate local cellular dynamics into whole-organ dynamics while accounting for the anisotropic properties of the myocardium (stimulus propagation and contraction). Rule-based methods have been developed for both the atria^19–22^ and the ventricles^21,23,24^ to incorporate sparse information from histology samples. Alternatively, universal coordinates, describing the position of a point within the heart independently of cardiac anatomy, can be used to map myofibre information from ex-vivo diffusion tensor MRI datasets^19^. The discretised, patient-specific anatomical model complete of local myofibre orientation can be used to run whole-organ simulations.

Propagation of the electrical stimulus within the cardiac tissue can be modelled with using three main types of formulations: the bidomain equations^25,26^, the monodomain equations^26^ or a reaction-Eikonal model^27^. The bidomain model is the most complex and detailed formulation available, where the myocardium is treated as a continuous medium with two interpenetrating domains, the intracellular and the extracellular spaces. The model provides the local distribution of the extracellular potential and the transmembrane potential, given parameters for local conductivities in the two domains. The cardiac anatomy can then be incorporated within a torso model to simulate propagation from the heart to the surrounding organs, to simulate surface signals and generate a 12-lead electrocardiogram (ECG). While the bidomain model provides a highly detailed representation of the electrical phenomena of the heart, it is also very computationally expensive. The monodomain equation constitutes a simpler and computationally cheaper formulation, under the assumption that intracellular and extracellular domains have the same anisotropy ratio. Both the monodomain and the bidomain formulations require the computation of the transmembrane ionic current (the net movement of charge across the cell membrane), typically computed using an ionic cell action potential model (see Section 1), where the transmembrane potential values used in the cell scale model is calculated as part of the tissue scale model. This is how cellular models are linked to tissue models to create a *multi-scale electrophysiology model.* Finally, the Eikonal model constitutes an even more simplified and very computationally efficient formulation compared to the monodomain and the bidomain formulations. This model, rather than solving complex voltage equations, computes the local time the activation wave will propagate to different areas of the heart, given an initial stimulus and the conduction velocity (or propagation speed) of different tissue types. This type of model takes seconds rather than hours to solve, making it more suitable to clinical timescales, although it lacks biophysical details provided by the bidomain and the monodomain formulations. Recently, a reaction-Eikonal model has been developed that combines the Eikonal with either a bidomain or monodomain approach to simulate the local transmembrane potential at a fraction of the computational cost^27^. The values of the conductivities (bidomain and monodomain) or conduction velocities (reaction-Eikonal) can be estimated for a specific patient using non-invasive or invasive measurements of a patient heart’s electrical activity, for example using a 12-lead ECG, electroanatomical mapping data or fluorescence images collected from isolated hearts. In summary, multi-scale electrophysiology models represent how the electrical stimulus propagates within the heart while accounting for the underlying cellular electrical activity and can be used to investigate how changes at the cellular level contribute towards the risk of cardiac arrhythmias.

The electrophysiology models described above are typically solved on a static anatomy, meaning that the effect of cardiac motion is neglected. To account for cardiac motion, multi-scale electromechanical models can be used. In these models, the anisotropic mechanical response of cardiac tissue (stiffer in the fibre direction) is represented with a constitutive law, describing how the myocardium develops stress in response to deformation^28–30^. The active stress or tension leading to cardiac contraction is computed with a model for cell contraction (see Section 1), given the local strain and velocity of contraction from the whole-heart model. To provide a physiological representation of the preload and the afterload of the heart, the mechanics model can be coupled with a model for the circulatory system (see Section 3)^31^. Multi-scale electromechanics models provide a wide range of information, from local motion, stress and strain to global quantities and biomarkers derived from simulated pressure and volume transients over time (e.g., ejection fraction, peak pressure, end-diastolic and end-systolic volumes). Clinical data such as image-derived motion, strain or volume transients as well as pressure transients can be used to calibrate the model parameters or for validation.

The electrophysiology and the mechanics models can be coupled into three ways: phenomenological, weak and strong. In phenomenological coupling, a phenomenological model for cell contraction (see Section 1) is used, discarding the biophysical processes leading to cell contraction. In weak coupling, the electrophysiology model triggers the mechanics through the intracellular calcium transient, but the mechanics does not affect the electrical activity of the cell. Finally, in strong coupling, the local strain from the mechanic model changes the behaviour of stretch-activated channels (mechano-electric feedback)^32^. Whole-heart electromechanics models provide a detailed representation of the heart’s electrical activity and mechanical contraction, compute clinically relevant biomarkers for cardiac function and, importantly, link them to electrical and mechanical function of cardiac myocytes.

# Blood flow models

Blood flow models aim at simulating blood flowing through cardiac tissue, large vessels, or the whole circulatory system. Based on this, these models can be divided into three categories: *perfusion models*, *computational fluid dynamics* (CFD) and *circulatory system models* (Fig. 1, bottom panel).

Cardiac perfusion models treat the myocardium as a porous medium, therefore lumping the small vessels (arterioles and capillaries) together in a continuum approximation with given permeability properties. These models do not simulate the blood flow in a particular capillary, but compute the distribution of blood flow in different regions of the heart, given the pressure of the coronary artery and venous resistance, supplying and extracting blood from a particular region of the heart, using the Darcy’s law^33–36^. Perfusion models can be parametrised and/or validated using data from perfusion imaging from MRI or CT or invasive measurements for fractional flow reserve. In summary, these models describe local perfusion of the myocardium and can provide information about how the latter changes in response to an occlusion or changes in the coronary microvasculature.

CFD simulates local blood flow dynamics within a vessel or a network of vessels. The vessels’ anatomy is segmented from imaging data (MRI, CT or angiograms), discretised, and used to solve a model describing the motion of a viscous fluid within the vessel (Navier-Stokes equations). This model computes the local velocity, pressure and shear stress provided an initial blood velocity profile or pressure at the vessel’s inlet and outlet, informing the model on how the fluid enters and exists the vessel, and the boundary conditions at the vessels’ wall. The vessels’ wall can be static, therefore requiring no information about the vessels’ motion, or can move over time. In this case, the motion can be derived from CT or MRI imaging data^37^, or from a mechanics model^38^ (see Section 2). When applied to the coronary tree, CFD can be used to provide inlet pressure for perfusion models^33^ (described above) and vice-versa, perfusion models can provide the outlet pressure for different coronary arteries. Coupling a CFD model of the coronary tree with a perfusion model links blood flow through large vessels with myocardial perfusion, which can be important to quantify the functional effects of a coronary occlusion.

Circulatory system models provide a simplified representation of the cardiovascular system, and they are often referred to as *lumped parameter models.* This type of framework assumes that each compartment (e.g., systemic or pulmonary arteries and veins, peripheral circulation) behaves as a simplified circuit with different properties of resistance, compliance and inertia, while pressure and flow are simulated as the voltage and the electrical current flowing through the system^39^. The heart is typically represented by a time-varying elastance model to account for its periodic contractile function. Clinical data for pressure, volumes and flows can be used to calibrate and/or validate the model. This more simplified representation is very computationally efficient and provides pressure and volume transients for the heart and different compartments of the circulatory system. However, it lacks detailed local representation of flow, pressure and strain dynamics provided by more complex 3D formulations.

One of the advantages of lumped parameter models is their flexibility, as a compartment can be replaced with a more detailed representation while the rest of the circulatory system provides physiological boundary conditions. As described in Section 2, circulatory system models can be coupled with a whole heart electromechanics framework, where the varying elastance model of the heart is replaced by a complex 3D formulation^40^. In this case, the circulatory system provides information about how the blood flows out of the ventricles and into the atria, and the whole-heart model provides inlet and outlet pressure for the arteries and the veins, respectively. Alternatively, a circulatory system model can be used to provide information at the boundaries of a coronary artery CFD model through aortic flow at the inlet and pressure at the outlets^33^. The flexibility of lumped parameter models makes them suitable for a wide range of applications and, when used in isolation, their computational efficiency makes them compatible with clinical timescales. As described in the manuscript, lumped parameter models have been used to performed *in-silico trials*, where computational modelling techniques are used to mirror a clinical trial to non-invasively test a treatment on a virtual patient population of interest.

In this Supplement, we have summarised different types of computational models of the heart and the circulatory system. This aimed at providing additional details that may be useful to understand the models described in the main manuscript, applied in the context of diabetes and anti-diabetic treatment.

# References

1. Karlstädt, A. *et al.* CardioNet: A human metabolic network suited for the study of cardiomyocyte metabolism. *BMC Syst Biol* 6, (2012).

2. Cortassa, S. *et al.* Metabolic remodelling of glucose, fatty acid and redox pathways in the heart of type 2 diabetic mice. *Journal of Physiology* 598, (2020).

3. Berndt, N. *et al.* CARDIOKIN1: Computational Assessment of Myocardial Metabolic Capability in Healthy Controls and Patients with Valve Diseases. *Circulation* 144, (2021).

4. Cortassa, S., Sollott, S. J. & Aon, M. A. Mitochondrial respiration and ROS emission during β-oxidation in the heart: An experimental-computational study. *PLoS Comput Biol* 13, (2017).

5. Jarosz, J. *et al.* Changes in mitochondrial morphology and organization can enhance energy supply from mitochondrial oxidative phosphorylation in diabetic cardiomyopathy. *Am J Physiol Cell Physiol* 312, (2017).

6. Ghosh, S. *et al.* Effects of altered cellular ultrastructure on energy metabolism in diabetic cardiomyopathy: an in silico study. *Philosophical Transactions of the Royal Society B: Biological Sciences* 377, (2022).

7. Tomek, J. *et al.* Development, calibration, and validation of a novel human ventricular myocyte model in health, disease, and drug block. *Elife* 8, (2019).

8. Courtemanche, M., Ramirez, R. J. & Nattel, S. Ionic mechanisms underlying human atrial action potential properties: Insights from a mathematical model. *Am J Physiol Heart Circ Physiol* 275, (1998).

9. Morotti, S. *et al.* Intracellular na+ modulates pacemaking activity in murine sinoatrial node myocytes: An in silico analysis. *Int J Mol Sci* 22, (2021).

10. Shannon, T. R., Wang, F., Puglisi, J., Weber, C. & Bers, D. M. A mathematical treatment of integrated Ca dynamics within the ventricular myocyte. *Biophys J* 87, (2004).

11. O’Hara, T., Virág, L., Varró, A. & Rudy, Y. Simulation of the undiseased human cardiac ventricular action potential: Model formulation and experimental validation. *PLoS Comput Biol* 7, (2011).

12. Pandit, S. V., Giles, W. R. & Demir, S. S. A mathematical model of the electrophysiological alterations in rat ventricular myocytes in type-I diabetes. *Biophys J* 84, (2003).

13. Land, S. *et al.* A model of cardiac contraction based on novel measurements of tension development in human cardiomyocytes. *J Mol Cell Cardiol* 106, (2017).

14. Lewalle, A., Milburn, G., Campbell, K. S. & Niederer, S. A. Cardiac length-dependent activation driven by force-dependent thick-filament dynamics. *Biophys J* (2024) doi:10.1016/j.bpj.2024.05.025.

15. Rice, J. J., Wang, F., Bers, D. M. & De Tombe, P. P. Approximate model of cooperative activation and crossbridge cycling in cardiac muscle using ordinary differential equations. *Biophys J* 95, (2008).

16. Niederer, S. A. *et al.* Length-dependent tension in the failing heart and the efficacy of cardiac resynchronization therapy. *Cardiovasc Res* 89, (2011).

17. Liu, T., Li, X., Wang, Y., Zhou, M. & Liang, F. Computational modeling of electromechanical coupling in human cardiomyocyte applied to study hypertrophic cardiomyopathy and its drug response. *Comput Methods Programs Biomed* 231, (2023).

18. Crozier, A. *et al.* Image-Based Personalization of Cardiac Anatomy for Coupled Electromechanical Modeling. *Ann Biomed Eng* 44, (2016).

19. Roney, C. H. *et al.* Constructing a Human Atrial Fibre Atlas. *Ann Biomed Eng* 49, (2021).

20. Roney, C. H. *et al.* Universal atrial coordinates applied to visualisation, registration and construction of patient specific meshes. *Med Image Anal* 55, (2019).

21. Piersanti, R. *et al.* Modeling cardiac muscle fibers in ventricular and atrial electrophysiology simulations. *Comput Methods Appl Mech Eng* 373, (2021).

22. Labarthe, S. *et al.* A bilayermodel of human atria:mathematical background, construction, and assessment. *Europace* 16, (2014).

23. Bayer, J. D., Blake, R. C., Plank, G. & Trayanova, N. A. A novel rule-based algorithm for assigning myocardial fiber orientation to computational heart models. *Ann Biomed Eng* 40, (2012).

24. Doste, R. *et al.* A rule-based method to model myocardial fiber orientation in cardiac biventricular geometries with outflow tracts. *Int J Numer Method Biomed Eng* 35, (2019).

25. Vigmond, E. J., Weber dos Santos, R., Prassl, A. J., Deo, M. & Plank, G. Solvers for the cardiac bidomain equations. *Progress in Biophysics and Molecular Biology* vol. 96 Preprint at https://doi.org/10.1016/j.pbiomolbio.2007.07.012 (2008).

26. Bishop, M. J. & Plank, G. Bidomain ECG simulations using an augmented monodomain model for the cardiac source. *IEEE Trans Biomed Eng* 58, (2011).

27. Neic, A. *et al.* Efficient computation of electrograms and ECGs in human whole heart simulations using a reaction-eikonal model. *J Comput Phys* 346, (2017).

28. Guccione, J. M., McCulloch, A. D. & Waldman, L. K. Passive material properties of intact ventricular myocardium determined from a cylindrical model. *J Biomech Eng* 113, (1991).

29. Holzapfel, G. A. & Ogden, R. W. Constitutive modelling of passive myocardium: A structurally based framework for material characterization. *Philosophical Transactions of the Royal Society A: Mathematical, Physical and Engineering Sciences* 367, (2009).

30. Usyk, T. P., Mazhari, R. & McCulloch, A. D. Effect of laminar orthotropic myofiber architecture on regional stress and strain in the canine left ventricle. *J Elast* 61, (2000).

31. Augustin, C. M. *et al.* A computationally efficient physiologically comprehensive 3D–0D closed-loop model of the heart and circulation. *Comput Methods Appl Mech Eng* 386, (2021).

32. Timmermann, V. *et al.* An integrative appraisal of mechano-electric feedback mechanisms in the heart. *Prog Biophys Mol Biol* 130, (2017).

33. Menon, K. *et al.* Personalized coronary and myocardial blood flow models incorporating CT perfusion imaging and synthetic vascular trees. *npj Imaging* 2, (2024).

34. Cookson, A. N. *et al.* A spatially-distributed computational model to quantify behaviour of contrast agents in MR perfusion imaging. *Med Image Anal* 18, (2014).

35. Zingaro, A., Vergara, C., Dede’, L., Regazzoni, F. & Quarteroni, A. A comprehensive mathematical model for cardiac perfusion. *Sci Rep* 13, (2023).

36. Di Gregorio, S. *et al.* A computational model applied to myocardial perfusion in the human heart: From large coronaries to microvasculature. *J Comput Phys* 424, (2021).

37. Karabelas, E. *et al.* Global Sensitivity Analysis of Four Chamber Heart Hemodynamics Using Surrogate Models. *IEEE Trans Biomed Eng* 69, (2022).

38. Santiago, A. *et al.* Fully coupled fluid-electro-mechanical model of the human heart for supercomputers. *Int J Numer Method Biomed Eng* 34, (2018).

39. Walmsley, J. *et al.* Fast Simulation of Mechanical Heterogeneity in the Electrically Asynchronous Heart Using the MultiPatch Module. *PLoS Comput Biol* 11, (2015).

40. Strocchi, M. *et al.* Cell to whole organ global sensitivity analysis on a four-chamber heart electromechanics model using Gaussian processes emulators. *PLoS Comput Biol* 19, (2023).
