# Supplementary material for: Cardiac digital twins: a tool to investigate the function and treatment of the diabetic heart
Source: Cardiovasc Diabetol. 2025 Jul 18;24:293. doi: 10.1186/s12933-025-02839-w (PMC12275252; doi:10.1186/s12933-025-02839-w)
Supplement: Supplementary file 2 — Supplementary material 2. [file 12933_2025_2839_MOESM2_ESM.docx]

| **Study** | **Species** | **Type of model** | **Treatment** | **Main conclusion** |  |
| --- | --- | --- | --- | --- | --- |
| **Metabolism models** | | | | | |
| *Zhou et al 2006* | - | Cardiac energy metabolism (cytosol + mitochondria) | - | Diabetes impairs the heart’s ability to adapt to increased energy demand |  |
| *Edwards et al 2011* | - | Oxidative phosphorylation model in the mitochondria and in the cytosol | - | Mitochondria abnormalities can contribute towards low PCr/ATP, especially when combined with hypoxia |  |
| *Cortassa et al 2017* | Guinea pig | Mitochondrial respiration model | - | High lipid concentrations impair mitochondrial function and lead to inefficient energy production and supply |  |
| *Cortassa et al 2020* | Mouse | Glucose catabolism and fatty acids oxidation in cytosol and mitochondria | Palmitate (fatty acid) | Glucose degradation pathways are ~2-fold lower in diabetic vs non-diabetic hearts. Palmitate treatment decreases glucose uptake and can improve metabolism of the diabetic heart |  |
| *Jarosz et al 2017* | Rat | Mitochondria model for energy production and metabolism | - | Diabetic cells had smaller but more dense mitochondria clustered together, serving as a compensatory mechanism to increase ATP availability for when ATP production is compromised |  |
| *Ghosh et al 2022* | Rat | Mitochondria model for energy production and metabolism in a finite element mesh of a 2D slice of a myocyte + mitochondria | - | Irregular arrangement of myofibril and mitochondria in diabetic cells impairs metabolite transport, leading to low energy availability in the myofibrils |  |
| **Action potential and calcium handling models** | | | | | |
| *Bassil et al 2018* | Mouse | Network model to link sympathetic and parasympathetic activity to the sinoatrial node | - | In diabetes, the pulmonary vein ganglia activity is reduced, potentially explaining increased risk of abnormal heart rhythm in diabetic patients |  |
| *Morotti et al 2021* | Mouse and human | Sinoatrial node action potential model | - | Increased intracellular sodium, typical of diabetic myocytes, alters automaticity and firing rate |  |
| *Bernjak et al 2021* | Human | Action potential model of the sinoatrial node with sympathetic or parasympathetic stimulation | - | Hypoglycaemia and hypokalaemia lead to a decrease in heart rate, especially when parasympathetic simulation is activated |  |
| *Fouda et al 2020* | Human | Action potential model of ventricular myocytes | Cannabidiol (CBD) | Glucose leads to concentration dependent APD prolongation through alteration of the sodium channel activation. CBD can protect against this effect |  |
| *Fouda et al 2021* | Human | Action potential model of ventricular myocytes | CBD and estradiol | CBD and importantly estradiol can protect against prolonged APD caused by glucose, highlighting the importance of female sex hormones in modulating sex-specific arrhythmia risk |  |
| *Ashrafi et al 2017* | Human | Action potential model of ventricular myocytes | - | Diabetic cells had longer APD and increased risk of early afterdepolarisation |  |
| *Pandit et al 2003* | Rat | Action potential models of the right ventricle | - | Diabetic cells had APD prolongation, reduced upstroke velocity and abnormal calcium handling |  |
| *Yaras et al 2005* | Rat | Action potential model of the papillary muscles | Selenium | Diabetic cells had longer APD. Selenium improved sodium-calcium exchanger function, leading to shorter APD |  |
| *Das et al 2017* | - | Model for calcium handling, glucose and GLUT4 | - | GLUT4 dysfunction causes abnormal oscillations in intracellular calcium |  |
| *Das et al 2020* | - | Model for the interaction between glucose, insulin and calcium handling | - | The time taken to transport glucose inside the cell through GLUT4 play an important role in maintaining physiological calcium handling |  |
| *Stewart et al 2018* | Rat | Model for calcium handling in a myocyte | - | Amylin (hormone released by the pancreas to regulate blood sugar) leads to increased calcium transients and calcium overload through increased permeability to calcium |  |
| *Op Den Bujis et al 2005* | Rat | Model of calcium fluxes in a cardiac myocyte | Isoprotenerol | Diabetic cells response to beta-adrenergic stimulation is smaller compared to controls due to differences in SERCA function |  |
| *Op Den Bujis et al 2008* | Rat | Cross-bridge cycling model | Isoprotenerol | The change in calcium and contractility following beta-adrenergic stimulation was blunted in the diabetic compared to the healthy heart |  |
| *Morotti et al 2017* | Rat | Arterial myocyte model for calcium and electrophysiology dynamics | - | L-type calcium channels are the primary determinant in changes in arterial myocyte dynamics due to high extracellular glucose |  |
| **Blood flow models** | | | | | |
| *Wang et al 2024* | Human | 1D-0D arterial network with a focus on renal circulation | - | Biomarkers for renal blood flow can help differentiate between diabetic nephropathy and hypertension |  |
| *Tunedal et al 2023* | Human | Lumped parameter model of the left side of the circulation | - | Patients with hypertension and type 2 diabetes, but not those with one or the other, had impaired LV relaxation |  |
| *Kulkarni et al 2018* | Human | Lumped parameter model for foetal circulation | - | In diabetic mothers, the blood flow is redistributed away from the brain of the foetus towards the placenta |  |
| *Branigan et al 2011* | - | Compliance model of vessels and model for pressure vs haematocrit | - | Blood pressure depends on the interaction between blood viscosity and blood vessel mechanical properties |  |
| *Ahmed et al 2024* | Human | Computational fluid dynamics of the carotid artery | - | The blood velocity in the carotid artery is decreased in diabetics, while the arterial pressure and wall shear increase |  |
| *Samyn et al 2015* | Human (children) | Computational fluid dynamics of the aorta | - | Wall shear stress was different between diabetics and controls, even though global measure of MRI-derived aortic distensibility was not different |  |
| *Xie et al 2023* | - | Model of plaque formation in hyperglycaemia and high cholesterol | - | The plaque persists in the presence of hyperglycaemia even at normal levels of low-density cholesterol and high-density cholesterol |  |
| *Behir et al 2024* | - | 2D computational fluid dynamics model of a cylindrical vessel with stenosis | - | Haematocrit, which is increased in diabetes, leads to changes in the local blood velocity and shear stress through a stenotic artery |  |
| *Luo et al 2023* | Human | Shape model of the retinal circulation | - | There is a correlation between retinal vascular shape and progression of diabetic retinopathy |  |
| *Wu et al 2022* | Human | Computational fluid dynamics of the ophthalmic artery | - | The diameter of the ophthalmic artery was smaller in diabetic vs non-diabetic patients with acute coronary syndrome; The pressure was higher and the velocity smaller in diabetic patients; Mass flow ratios of the ophthalmic artery correlated with NT-proBNP |  |
| *Bernabeu et al 2018* | Human | Retinal microvasculature | - | Areas with low shear rate are associated with clotting in saccular micro-aneurisms |  |
| **In-silico trials** | | | | | |
| *Hallow et al 2018* | Human | Model to link diuresis and sodium clearance to blood and interstitial fluid | Diuretics and dapagliflozin | Dapagliflozin leads to a greater interstitial volume reduction vs interstitial fluid volume reduction compared to diuretics, suggesting that it could provide better control of congestion without reducing arterial filling and perfusion |  |
| *Hallow et al 2018* | Human | Lumped parameter model for renal circulation | Dapagliflozin | Diuresis and NHE3 inhibition led to reduced glomerular pressure, reduced blood volume, clearance of interstitial fluids and mild blood pressure reduction, and these effects are more significant in diabetic patients compared to controls |  |
| *Yu et al 2022* | Human | Myocyte and heart model coupled with a lumped model for the circulatory system and renal vasculature | Dapagliflozin | Strains and cardiac efficiency do not improve with dapagliflozin in patients without heart failure, but they do in patients with heart failure with reduced ejection fraction |  |
| *Yu et al 2021* | Human | Myocyte and heart model coupled with a lumped model for the circulatory system and renal vasculature | Dapagliflozin | The renal effects of dapagliflozin leads to decreased sodium and water retention leading to reduced blood volume and therefore left ventricular end-diastolic volume and pressure |  |
| *Yu et al 2024* | Human | Lumped parameter model for renal circulation and renal function | Renin-angiotensin-aldosterone inhibitors and diuretics | Venodilation and increased venous capacitance through endothelin-1 receptor A antagonists was necessary to reproduce the reduced blood pressure and haematocrit observed in the data |  |

**Supplementary table.** Summary of all computational studies included in the manuscript investigating the effect of diabetes and of anti-diabetic treatment of the heart. For each study, we show the first author and year, the species the model was built for, if available, the type of model, the drug treatment included in the study if any, and the main conclusion of the study.
